# Alagille syndrome mutation update: Comprehensive overview of *JAG1* and *NOTCH2* mutation frequencies and insight into missense variant classification

**DOI:** 10.1002/humu.23879

**Published:** 2019-08-26

**Authors:** Melissa A. Gilbert, Robert C. Bauer, Ramakrishnan Rajagopalan, Christopher M. Grochowski, Grace Chao, Deborah McEldrew, James A. Nassur, Elizabeth B. Rand, Bryan L. Krock, Binita M. Kamath, Ian D. Krantz, David A. Piccoli, Kathleen M. Loomes, Nancy B. Spinner

**Affiliations:** ^1^ Division of Genomic Diagnostics, Department of Pathology and Laboratory Medicine, Children's Hospital of Philadelphia and The Perelman School of Medicine University of Pennsylvania Philadelphia Pennsylvania; ^2^ Division of Pediatric Gastroenterology, Hepatology, and Nutrition, Department of Pediatrics, Children's Hospital of Philadelphia and The Perelman School of Medicine University of Pennsylvania Philadelphia Pennsylvania; ^3^ Division of Gastroenterology, Hepatology and Nutrition, Department of Pediatrics Hospital for Sick Children and the University of Toronto Toronto Canada; ^4^ Division of Human Genetics, Roberts Individualized Medical Genetics Center Children's Hospital of Philadelphia Philadelphia Pennsylvania; ^5^ Department of Pediatrics The Perelman School of Medicine at the University of Pennsylvania Philadelphia Pennsylvania

**Keywords:** Alagille syndrome, JAG1, liver, NOTCH2

## Abstract

Alagille syndrome is an autosomal dominant disease with a known molecular etiology of dysfunctional Notch signaling caused primarily by pathogenic variants in *JAGGED1* (*JAG1*), but also by variants in *NOTCH2*. The majority of *JAG1* variants result in loss of function, however disease has also been attributed to lesser understood missense variants. Conversely, the majority of *NOTCH2* variants are missense, though fewer of these variants have been described. In addition, there is a small group of patients with a clear clinical phenotype in the absence of a pathogenic variant. Here, we catalog our single‐center study, which includes 401 probands and 111 affected family members amassed over a 27‐year period, to provide updated mutation frequencies in *JAG1* and *NOTCH2* as well as functional validation of nine missense variants. Combining our cohort of 86 novel *JAG1* and three novel *NOTCH2* variants with previously published data (totaling 713 variants), we present the most comprehensive pathogenic variant overview for Alagille syndrome. Using this data set, we developed new guidance to help with the classification of *JAG1* missense variants. Finally, we report clinically consistent cases for which a molecular etiology has not been identified and discuss the potential for next generation sequencing methodologies in novel variant discovery.

## 1 BACKGROUND

Alagille syndrome (ALGS; MIM# 118450) is an autosomal dominant disorder with an incidence of 1:30,000 to 1:50,000 live births, that was first described in the early 1970 s based on the clinical observation of characteristic liver, cardiac, eye, vertebral, and facial phenotypes (Alagille, Odievre, Gautier, & Dommergues, 1975; Crosnier, Lykavieris, Meunier‐Rotival, & Hadchouel, 2000; Emerick et al., 1999; Saleh, Kamath, & Chitayat, 2016; Spinner et al., 2001; Watson & Miller, 1973). Fine mapping chromosome 20p12 in several patients with ALGS led to the identification of pathogenic variants in the Notch signaling ligand, *JAGGED1* (*JAG1*) as disease‐causing (Byrne, Harrod, Friedman, & Howard‐Peebles, 1986; Krantz et al., 1997; Li et al., 1997; Oda et al., 1997; Pollet et al., 1997; Spinner et al., 1994). Since then, the molecular etiology of the disease has been defined by Notch signaling dysfunction, and pathogenic variants in the Notch signaling receptor *NOTCH2* have also been identified, although they are found less frequently than those in *JAG1* (Kamath et al., 2012; McCright, Lozier, & Gridley, 2002; McDaniell et al., 2006).

JAG1 and NOTCH2 are both single‐pass transmembrane proteins, consisting of 26 and 34 exons, respectively. Direct communication between the two proteins is accomplished through interaction of the extracellular domain of JAG1 (ligand) with NOTCH2 (receptor). Numerous functional motifs are required for this interaction, including the delta‐serate‐lag2 (DSL) domain, the C2‐like domain, and the epidermal growth factor‐like (EGF‐like) repeats on JAG1 and extracellular EGF‐like repeats located on NOTCH2 (Chillakuri et al., 2013; Kopan & Ilagan, 2009; Lindsell, Shawber, Boulter, & Weinmaster, 1995). NOTCH2 also contains a series of Ankyrin (ANK) repeats, which are required for signal propagation and allow for the interaction of the intracellular region of NOTCH2 with transcription factors (Tamura et al., 1995). There have been many recent reviews on ALGS, *JAG1*, and *NOTCH2* that we recommend for additional reference (Bray, 2016; Gilbert & Spinner, 2017; Grochowski, Loomes, & Spinner, 2016; Saleh et al., 2016).

Pathogenic variants in *JAG1* are most commonly protein‐truncating, including frameshift, nonsense, exon level deletions, and splice site, though missense variants and whole gene deletions have also been reported (Crosnier et al., 1999; Warthen et al., 2006). The predominance of these protein‐truncating variants along with the observation that both whole gene deletions and intragenic pathogenic variants cause similar phenotypes, supports a haploinsufficient disease mechanism (Oda et al., 1997; Saleh et al., 2016; Spinner et al., 2001). Early studies aimed to determine whether the location of pathogenic variants is able to predict the clinical manifestation of the disease do not support a genotype‐phenotype correlation (Crosnier et al., 1999; Spinner et al., 2001). Conversely, a high degree of variable expressivity has been observed, and often significant phenotypic variability is reported in families harboring the same pathogenic variant (Dhorne‐Pollet, Deleuze, Hadchouel, & Bonaiti‐Pellie, 1994; Elmslie et al., 1995; Emerick et al., 1999; Izumi et al., 2016; Kamath, Bason, Piccoli, Krantz, & Spinner, 2003; Kamath, Krantz, Spinner, Heubi, & Piccoli, 2002; Krantz et al., 1998; Shulman, Hyams, Gunta, Greenstein, & Cassidy, 1984). These observations have led to the hypothesis that a second gene could act as a modifier, and studies have been carried out to test this theory. It has been proposed that defects in glycosylation of the mature JAG1 and NOTCH2 proteins will result in mutant proteins that are improperly trafficked and not effectively expressed at the cell membrane. Lunatic Fringe, Radical Fringe, Manic Fringe, and POGLUT1 are all known glycosyltransferases that have been studied in this capacity, and data is supportive of a role for these proteins in modifying the effects of pathogenic *JAG1* variants (Ryan et al., 2008; Thakurdas et al., 2016). A second candidate genetic modifier, *THROMBOSPONDIN2* (*THBS2*), was identified from a Genome Wide Association Study (GWAS) that stratified ALGS patients with pathogenic variants in *JAG1* by whether they had mild or severe liver disease (Tsai et al., 2016). *THBS2* encodes an extracellular matrix protein that is expressed in murine bile ducts and can interact with Notch signaling. Data from the GWAS study suggested that individuals with a pathogenic *JAG1* variant and increased *THBS2* expression could be at risk for developing more severe liver disease (Tsai et al., 2016).

The pathogenic mechanism of *NOTCH2* variants has been far less clear than with *JAG1*. Fewer pathogenic *NOTCH2* variants have been identified, and unlike with *JAG1*, these variants are predominantly missense (Kamath et al., 2012). It is possible that *NOTCH2* is less tolerant than *JAG1* to missense variants, resulting in functional haploinsufficiency, however other mechanisms of pathogenesis may be in effect. The higher frequency of missense variants in *NOTCH2* may also indicate that *NOTCH2* is intolerant of more severe, loss of function variants. As with pathogenic *JAG1* variants, genotype–phenotype correlations have not been noted with *NOTCH2* variants, though very few patients with *NOTCH2* variants have been described to date. However, it has been reported on a preliminary basis that the clinical presentation of individuals with pathogenic *NOTCH2* variants is different from those with pathogenic *JAG1* variants, with a lower prevalence of cardiac involvement, vertebral anomalies, and facial features (Kamath et al., 2012).

In 1997, before the discovery that pathogenic variants in *JAG1* cause ALGS, our lab initiated a clinical study to identify the causal gene for ALGS. Because that time, we had enrolled 401 probands who are clinically‐consistent with ALGS, as well as numerous affected and unaffected relatives to test for inheritance. We and others have previously described 608 *JAG1* variants and 16 *NOTCH2* variants that are thought to cause disease (Fokkema et al., 2011; Landrum et al., 2018; Stenson et al., 2017). Here, we report an additional 86 novel *JAG1* and three novel *NOTCH2* pathogenic variants, and provide functional validation for nine previously uncharacterized *JAG1* missense variants. Through this mutation update, we aim to combine our data of 27 years with previously published data of known pathogenic and likely pathogenic variants to provide up‐to‐date statistics on the frequency and type of *JAG1* and *NOTCH2* variants in ALGS. In addition, we will discuss mutation trends that we and others have observed in both the *JAG1* and *NOTCH2* genes as a resource for missense variant interpretation and classification. Finally, we will end with our thoughts on how best to understand the small population of patients with clinically defined ALGS who do not have a pathogenic variant in *JAG1* or *NOTCH2* and are currently molecularly uncharacterized.

## 2 MATERIALS AND METHODS

### 2.1 Patient cohort

We studied 401 probands whose phenotypic features met the clinical definition of ALGS based on the presence of three out of five characteristic liver, heart, eye, vertebral, and/or facial phenotypes as previously described (Alagille et al., 1987; Emerick et al., 1999; Kamath et al., 2003). The majority of these probands were ascertained from the Liver Clinic at the Children's Hospital of Philadelphia (CHOP), therefore, enriching our patient population for liver disease and potentially for *JAG1* pathogenic variants associated with cholestasis. We also include data from 111 affected family members.

Some of the patients in our cohort have been previously reported and are included here to provide a comprehensive summary of our clinical study, with prior reports referenced in all corresponding tables (Bauer et al., 2010; Colliton et al., 2001; Heritage et al., 2000; Izumi et al., 2016; Kamath et al., 2003; Kamath et al., 2009; Kamath et al., 2012; Krantz et al., 1998; Laufer‐Cahana et al., 2002; Li et al., 1997; Lin et al., 2012; McDaniell et al., 2006; Morrissette, Colliton, & Spinner, 2001; Oda et al., 1997; Warthen et al., 2006). Our cohort contains both probands and affected family members. All patients were enrolled into our study using a consent protocol approved by the Institutional Review Board at CHOP. All *JAG1* variants described in our study can be retrieved from an already existing Locus Specific Database (LSDB) using the following link: https://databases.lovd.nl/shared/genes/JAG1.

### 2.2 Literature search

The majority of reported *JAG1* and *NOTCH2* variants are found in The Human Gene Mutation Database (HGMD® Professional 2019.1, last queried on May 3, 2019; Stenson et al., 2017). Variants were filtered to include only those that were reported to be disease‐causing (“DM”) and were associated with ALGS. Variants were also identified from ClinVar (last queried on May 3, 2019), and were filtered to include only those that were reported as “pathogenic” or “likely pathogenic” and listed “Alagille syndrome” as the associated condition (Landrum et al., 2018). A literature search was also performed on PubMed, with a last check on May 3, 2019. Finally, Leiden Open Variation Database (LOVD V3.0) was last queried on May 3, 2019 for *JAG1*, and variants were filtered to include only those reported as “pathogenic” or “likely pathogenic” (Fokkema et al., 2011).

### 2.3 Mutation identification

Genomic DNA extracted from whole blood was screened first by polymerase chain reaction (PCR) and Sanger sequencing of all 26 exons of the *JAG1* gene. Samples in which no pathogenic or likely pathogenic variant was identified were further screened by MLPA or single nucleotide polymorphism (SNP) array analysis of the *JAG1* gene to identify copy number variants. If a sample was not found to have a pathogenic or likely pathogenic variant in *JAG1* by both PCR and MLPA analysis, the sample was screened for pathogenic variants in the *NOTCH2* gene by PCR and Sanger sequencing. Patients who were diagnosed as clinically consistent with ALGS, but in whom no pathogenic or likely pathogenic variant was identified by this three‐tiered approach were classified as mutation‐negative. PCR‐free whole genome sequencing (150 bp paired‐end reads) at an average depth of 30× was performed using HiSeq X at the Center for Applied Genomics at the Children's Hospital of Philadelphia.

### 2.4 Mutant JAG1 constructs

Human *JAG1* cDNA has previously been cloned into the pBABE‐puro retroviral expression vector (Morrissette et al., 2001). Point mutations were introduced using the QuikChange Site‐Directed Mutagenesis Kit (Stratagene, San Diego, CA) and resultant clones were sequenced for mutation verification. Stable cell lines were generated by infecting NIH‐3T3 cells with these mutant retroviral vectors as previously described (Morrissette et al., 2001).

### 2.5 Enzymatic assays

Trypsin: Cells were treated with 2 ml of 0.25% trypsin in EDTA (Gibco, Gaithersburg, MD) at 37°C for 10 min before inactivation and protein extraction using NP40 lysis buffer (1% NP40, 150 mM NaCl, 50 mM Tris‐HCl, final pH 8.0) plus 1 μM DTT, 25 μM PMSF, and 0.1 μg/ml aprotinin and leupeptin.

Endo H: 50 micrograms of protein obtained from NP40 lysis were treated with 1,500 units of Endo H (New England Biolabs, Ipswitch, MA) at 37°C for 1 hr.

### 2.6 Western blot analysis

Western blot analysis was performed according to standard protocols. JAG1 was detected using an antibody recognizing the C‐terminal region (H‐114, Santa Cruz Biotechnology, Inc., Dallas, TX) and a HRP‐goat anti‐rabbit secondary antibody (Amersham, Inc. Buckinghamshire, United Kingdom).

### 2.7 Immunofluorescence

Stable cell lines were plated on culture slides and treated as previously described (Bauer et al., 2010). A JAG1 antibody (H‐114; Santa Cruz Biotechnology, Inc.) was used at a 1:40 dilution for immunodetection.

### 2.8 Luciferase assays

Luciferase assays were performed as previously described (Bauer et al., 2010). Briefly, cells transfected with 199 ng of 4xCBF‐Luc reporter construct (Hsieh et al., 1996) and 1 ng of an internal control SV40 *Renilla* construct (Promega, Madison, WI) were cocultured with stable cell lines expressing mutant *JAG1*. Firefly luciferase was normalized to *Renilla* luciferase and reported as fold change over pBABE alone. All experiments were performed in triplicate.

## 3 VARIANTS IN *JAG1*

### 3.1 Frequency and types of JAG1 pathogenic variants

**Figure 1:**
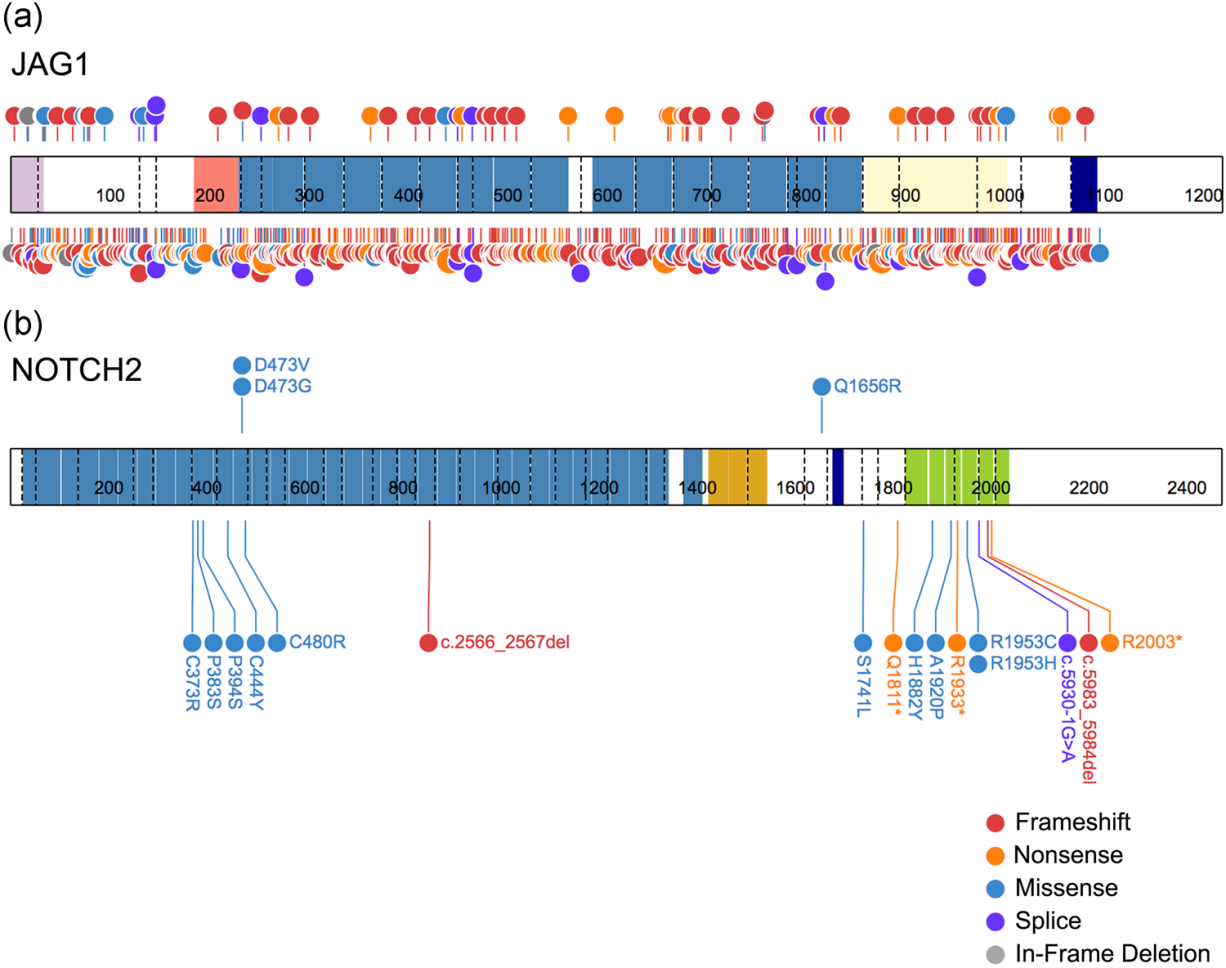
Schematic of JAG1 and NOTCH2 proteins with all reported and novel pathogenic variants. (a) JAG1 and (b) NOTCH2 proteins are depicted with all reported pathogenic variants shown below the schematic and all novel pathogenic variants reported here shown above the schematic. Dashed lines within the protein indicate exon boundaries and numbers indicate amino acid coordinates. Protein domains include (JAG1): signal peptide (lavender), DSL domain (salmon), EGF‐like repeats (blue), cysteine‐rich domain (yellow), and transmembrane domain (purple) and (NOTCH2): EGF‐like repeats (blue), LNR domain (yellow), transmembrane domain (purple), and ANK repeats (green). RefSeq NM_000214.2 (JAG1) and NM_024408.3 (NOTCH2). Images were prepared using ProteinPaint software from Saint Jude Children's Research Hospital–Pediatric Cancer Data Portal (Zhou et al., 2016)

We identified 297 unique *JAG1* pathogenic or likely pathogenic variants in 378 of 401 (94.3%) probands in our cohort (Tables 1, 2; Figure S1). These variants encompass frameshift (nucleotide‐level deletions, duplications, insertions, and insertion–deletions), nonsense (substitutions, start loss, stop gain), missense, splice site, in‐frame deletions, large gene deletions (single exon, multi‐exon, or full‐gene deletions), partial gene duplications (multi‐exon duplications), and complex rearrangements. Our list includes 86 novel pathogenic variants that have not previously been reported (Figure 1a). All 297 variants described in our study were submitted to the existing LSDB on *JAG1* (https://databases.lovd.nl/shared/genes/JAG1).

**Table 1:** *JAG1* pathogenic variants reported in our study

Exon/Intron	DNA variant	Protein change	Coding effect	Protein domain	Probands	Affected family members	Novel	Frequency in gnomAD	References	Remarks
1	c.3_4delinsTT	p.?	Start loss	SP	1	0	No	Not present	Colliton et al. (2001)	
1	c.11dup	p.Arg5Thrfs*68	Frameshift	SP	1	0	Yes	Not present		
1	c.50 T>C	p.Leu17Pro	Missense	SP	2	0	Yes	Not present		
1	c.53_73del	p.Leu18_Leu24del	In‐frame deletion	SP	1	0	Yes	Not present		
1	c.59 T>C	p.Leu20Pro	Missense	SP	1	0	No	Not present	Guegan, Stals, Day, Turnpenny, and Ellard (2012)	
1	c.59 T>G	p.Leu20Arg	Missense	SP	1	2	No	Not present	Izumi et al. (2016)	
1	c.62_73del	p.Leu21_Leu24del	In‐frame deletion	SP	1	3	No	Not present	Warthen et al. (2006)	Previously reported as c.63_74del; p.Cys22_Argdel
1	c.64 T>C	p.Cys22Arg	Missense	SP	2	0	No	Not present	Lin et al. (2012)	
1	c.66_67del	p.Ala23Profs*49	Frameshift	SP	1	0	No	Not present	Colliton et al. (2001)	
1	c.70del	p.Leu24Cysfs*22	Frameshift	SP	1	0	No	Not present	Colliton et al. (2001)	
Intron 1	c.81+1 G>A		Splice site		1	0	No	Not present	Warthen et al. (2006)	
2	c.97 G>A	p.Gly33Ser	Missense	C2‐like domain	1	0	No	Not present	Warthen et al. (2006)	
2	c.97 G>C	p.Gly33Arg	Missense	C2‐like domain	1	0	Yes	Not present		
2	c.98 G>A	p.Gly33Asp	Missense	C2‐like domain	1	0	No	Not present	Colliton et al. (2001)	
2	c.98 G>T	p.Gly33Val	Missense	C2‐like domain	1	0	No	Not present	Warthen et al. (2006)	
2	c.100_103del	p.Gln34Serfs*11	Frameshift	C2‐like domain	1	0	Yes	Not present		
2	c.104 T>C	p.Phe35Ser	Missense	C2‐like domain	1	2	Yes	Not present		
2	c.110 T>C	p.Leu37Ser	Missense	C2‐like domain	1	0	No	Not present	Colliton et al. (2001); Bauer et al. (2010)	
2	c.127 C>T	p.Gln43*	Nonsense	C2‐like domain	1	0	No	Not present	Colliton et al. (2001)	
2	c.139_152del	p.Gly47Argfs*21	Frameshift	C2‐like domain	1	0	No	Not present	Warthen et al. (2006)	
2	c.141_142del	p.Glu48Alafs*24	Frameshift	C2‐like domain	1	0	Yes	Not present		
2	c.148 C>T	p.Gln50*	Nonsense	C2‐like domain	1	1	No	Not present	Colliton et al. (2001)	
2	c.161_162delinsAA	p.Cys54*	Stop gain	C2‐like domain	1	0	No	Not present	Yuan et al. (2001)	
2	c.165 C>A	p.Cys55*	Nonsense	C2‐like domain	1	0	No	Not present	Warthen et al. (2006)	
2	c.187_188del	p.Asp63Profs*9	Frameshift	C2‐like domain	1	0	Yes	Not present		
2	c.211_225del	p.Cys71_Phe75del	In‐frame deletion	C2‐like domain	1	0	No	Not present	Colliton et al. (2001)	
2	c.221 A>G	p.Tyr74Cys	Missense	C2‐like domain	1	2	Yes	Not present		
2	c.232 T>A	p.Cys78Ser	Missense	C2‐like domain	1	0	Yes	Not present		
2	c.232 T>G	p.Cys78Gly	Missense	C2‐like domain	1	0	No	Not present	Lin et al. (2012)	
2	c.237dup	p.Lys80Glnfs*64	Frameshift	C2‐like domain	1	0	Yes	Not present		
2	c.266_270delinsCTT	p.Gly89Alafs*54	Frameshift	C2‐like domain	1	0	No	Not present	Warthen et al. (2006)	
2	c.270del	p.Cys92Alafs*69	Frameshift	C2‐like domain	1	0	No	Not present	Warthen et al. (2006); Agrawal, Chennuri, and Agrawal (2015)	
2	c.270dup	p.Pro91Alafs*53	Frameshift	C2‐like domain	2	1	No	Not present	Krantz et al. (1998); Krantz et al. (1999)	
2	c.274 T>C	p.Cys92Arg	Missense	C2‐like domain	1	0	No	Not present	Warthen et al. (2006)	
2	c.275 G>A	p.Cys92Tyr	Missense	C2‐like domain	1	3	No	Not present	Warthen et al. (2006)	
2	c.283 G>C	p.Gly95Arg	Missense	C2‐like domain	1	2	Yes	Not present		
2	c.287 C>A	p.Ser96*	Nonsense	C2‐like domain	1	0	No	Not present	Warthen et al. (2006)	
2	c.291del	p.Ser98Profs*63	Frameshift	C2‐like domain	1	0	No	Not present	Warthen et al. (2006)	
2	c.291_297del	p.Ser98Leufs*61	Frameshift	C2‐like domain	1	0	No	Not present	Warthen et al. (2006)	
2	c.311dup	p.Asn105Glnfs*39	Frameshift	C2‐like domain	1	0	No	Not present	Stalke et al. (2018)	
2	c.337dup	p.Arg113Profs*31	Frameshift	C2‐like domain	1	0	No	Not present	Lin et al. (2012)	
Intron 2	c.387+1 G>A		Splice site		1	0	No	Not present	Warthen et al. (2006)	
Intron 2	c.388–17_391del		Splice site		1	0	Yes	Not present		Unconfirmed by cDNA
Intron 2	c.388–1 G>C		Splice site		1	0	No	Not present	Jurkiewicz, Popowska, Glaser, Hansmann, and Krajewska‐Walasek (2005)	
3	c.401 T>C	p.Leu134Ser	Missense	C2‐like domain	1	0	Yes	Not present		
3/Intron 3	c.435_439+2delinsAG		Splice site		1	0	Yes	Not present		Unconfirmed by cDNA
3/Intron 3	c.438_439+2del	p.Val146Asnfs*14	Frameshift	C2‐like domain	1	1	No	Not present	Guegan et al. (2012)	cDNA shows utilization of a cryptic splice donor site at c.436_437 resulting in a frameshift
3	c.439 C>T	p.Gln147*	Nonsense	C2‐like domain	2	1	No	Not present	Warthen et al. (2006)	
Intron 3	c.439+1 G>T		Splice site		1	1	Yes	Not present		
Intron 3	c.439+1 G>A		Splice site		4	0	No	Not present	Crosnier et al. (2000); Ohashi et al. (2017)	
Intron 3	c.439+1 G>C		Splice site		1	0	Yes	Not present		
Intron 3	c.439+5 G>A	Deletion of Exon 3	Splice site		2	1	Yes	Not present		Confirmed by cDNA
Intron 3	c.439+6 T>A	Deletion of Exon 3	Splice site		1	0	No	Not present	Warthen et al. (2006)	Confirmed by cDNA
4	c.463 G>C	p.Ala155Pro	Missense	C2‐like domain	2	0	No	Not present	Warthen et al. (2006)	
4	c.488 C>G	p.Pro163Arg	Missense	C2‐like domain	1	0	No	Not present	Ropke, Kujat, Graber, Giannakudis, and Hansmann (2003)	
4	c.514 C>T	p.Gln172*	Nonsense	C2‐like domain	2	3	No	Not present	Colliton et al. (2001)	
4	c.518_521dup	p.Gly175Hisfs*7	Frameshift	C2‐like domain	1	0	No	Not present	Colliton et al. (2001)	
4	c.541 T>A	p.Tyr181Asn	Missense	C2‐like domain	1	0	No	Not present	Colliton et al. (2001)	
4	c.550 C>T	p.Arg184Cys	Missense	C2‐like domain	3	0	No	Not present	Krantz et al. (1998)	
4	c.551 G>A	p.Arg184His	Missense	C2‐like domain	7	1	No	Not present	Krantz et al. (1998); Tada et al. (2012)	
4	c.582dup	p.Gly195Trpfs*4	Frameshift	DSL	1	0	No	Not present	Warthen et al. (2006)	
4	c.625del	p.His209Thrfs*203	Frameshift	DSL	1	0	Yes	Not present		
4	c.659_661delinsTG	p.Cys220Leufs*192	Frameshift	DSL	1	0	No	Not present	Krantz et al. (1998)	
4	c.686 G>A	p.Cys229Tyr	Missense	DSL	1	0	No	Not present	Heritage et al. (2000)	
4	c.693_694del	p.Arg231Serfs*10	Frameshift	EGF1	4	3	No	Not present	Li et al. (1997)	
5	c.700 T>G	p.Cys234Gly	Missense	EGF1	1	0	Yes	Not present		
5	c.701del	p.Cys234Serfs*178	Frameshift	EGF1	1	1	Yes	Not present		
5	c.703 C>T	p.Arg235*	Nonsense	EGF1	6	1	No	Not present	Krantz et al. (1998)	
5	c.739_746del	p.Leu247*	Stop gain	EGF1	1	0	No	Not present	Warthen et al. (2006)	
5	c.754 A>G	p.Arg252Gly	Missense	EGF1	1	1	No	Not present	Warthen et al. (2006)	Predicted to change splice donor site
Intron 5	c.755+2 T>A		Splice site		1	2	No	Not present	Warthen et al. (2006)	
Intron 5	c.755+1_755+2dup	Deletion of Exon 5	Splice site		1	1	Yes	Not present		Confirmed by cDNA
6	c.765 C>G	p.Tyr255*	Nonsense	EGF1	2	0	No	Not present	Witt, Neumann, Grollmuss, Luck, and Becker (2004)	
6	c.766 G>A	p.Gly256Ser	Missense	EGF1	1	0	No	Not present	Warthen et al. (2006)	
6	c.794 G>T	p.Cys265Phe	Missense	EGF2	1	1	No	Not present	Guegan et al. (2012)	
6	c.808 G>T	p.Gly270*	Nonsense	EGF2	1	0	Yes	Not present		
6	c.811 T>C	p.Cys271Arg	Missense	EGF2	1	0	No	Not present	Warthen et al. (2006)	
6	c.838_839dup	p.Trp280Cysfs*133	Frameshift	EGF2	1	1	Yes	Not present		
6	c.839 G>A	p.Trp280*	Nonsense	EGF2	1	0	No	Not present	Lin et al. (2012)	
6	c.871 C>T	p.Gln291*	Nonsense	EGF2	1	1	No	Not present	Vazquez‐Martinez et al. (2014)	
Intron 6	c.886+1 G>T	Deletion of Exon 6	Splice site		2	2	No	Not present	Warthen et al. (2006)	Confirmed by cDNA
Intron 6	c.886+2 T>G		Splice site		1	1	No	Not present	Oda et al. (1997)	
7	c.903dup	p.Thr302Aspfs*12	Frameshift	EGF3	1	0	Yes	Not present		
7	c.910 C>T	p.Gln304*	Nonsense	EGF3	1	0	No	Not present	Warthen et al. (2006)	
7	c.918 T>A	p.Cys306*	Nonsense	EGF3	1	0	No	Not present	Krantz et al. (1998)	
7	c.986 C>A	p.Ser329*	Nonsense	EGF3	1	0	No	Not present	Warthen et al. (2006)	
8	c.1019_1022del	p.Cys340Serfs*71	Frameshift	EGF4	1	0	No	Not present	Lin et al. (2012)	
8	c.1057 G>T	p.Glu353*	Nonsense	EGF4	2	3	No	Not present	Colliton et al. (2001)	
8	c.1080_1081del	p.Cys360*	Stop gain	EGF4	1	0	No	Not present	Colliton et al. (2001)	
8	c.1086 T>A	p.Cys362*	Nonsense	EGF4	1	0	Yes	Not present		
9	c.1126del	p.Asp376Metfs*36	Frameshift	EGF5	1	0	No	Not present	Warthen et al. (2006)	
9	c.1139del	p.Pro380Leufs*32	Frameshift	EGF5	1	0	Yes	Not present		
9	c.1156 G>A	p.Gly386Arg	Missense	EGF5	4	3	No	Not present	Heritage et al. (2000); Tada et al. (2012)	
9	c.1189 A>T	p.Lys397*	Nonsense	EGF5	1	0	No	Not present	Krantz et al. (1998)	
9	c.1191del	p.Lys397Asnfs*15	Frameshift	EGF5	1	0	No	Not present	Colliton et al. (2001)	
9	c.1198_1199del	p.Cys400Profs*15	Frameshift	EGF5	1	0	No	Not present	Warthen et al. (2006)	
9	c.1205del	p.Pro402Hisfs*10	Frameshift	EGF5	1	0	No	Not present	Lin et al. (2012)	
9	c.1205dup	p.Gln403Thrfs*13	Frameshift	EGF5	3	0	No	Not present	Krantz et al. (1998)	
9	c.1207 C>T	p.Gln403*	Nonsense	EGF5	3	0	No	Not present	Krantz et al. (1998)	
9	c.1222dup	p.Thr408Asnfs*8	Frameshift	EGF5	1	0	Yes	Not present		
10	c.1242del	p.Asn414Lysfs*9	Frameshift	EGF6	1	2	No	Not present	Warthen et al. (2006)	
10	c.1264_1265del	p.Val422Lysfs*6	Frameshift	EGF6	1	0	Yes	Not present		
10	c.1296_1299dup	p.Tyr434Glnfs*16	Frameshift	EGF6	1	0	No	Not present	Krantz et al. (1998)	
10	c.1305 C>G	p.Tyr435*	Nonsense	EGF6	1	6	No	Not present	Colliton et al. (2001)	
10	c.1313 G>T	p.Cys438Phe	Missense	EGF6	1	0	No	Not present	Crosnier et al. (1999)	
10	c.1313 G>C	p.Cys438Ser	Missense	EGF6	1	0	Yes	Not present		
10	c.1325 G>A	p.Trp442*	Nonsense	EGF6	1	0	No	Not present	Warthen et al. (2006)	
10	c.1326 G>A	p.Trp442*	Nonsense	EGF6	1	1	No	Not present	Giannakudis et al. (2001); Ohashi et al. (2017)	
Intron 10	c.1349–12 T>G	Deletion of Exon 11	Splice site		1	1	No	Not present	Krantz et al. (1998)	confirmed by cDNA
Intron 10	c.1349–1 G>C		Splice site		2	0	Yes	Not present		
11	c.1362 C>A	p.Cys454*	Nonsense	EGF7	1	0	Yes	Not present		
11/Intron 11	c.1393_1395+2del		Splice site	EGF7	1	0	Yes	Not present		Unconfirmed by cDNA
Intron 11	c.1395+1 G>T		Splice site		2	0	No	Not present	Krantz et al. (1998)	
Intron 11	c.1395+1 G>A		Splice site		1	0	No	Not present	Warthen et al. (2006)	Confirmed by cDNA
12	c.1419dup	p.Ile474Tyrfs*12	Frameshift	EGF7	1	2	No	Not present	Guegan et al. (2012)	
12	c.1433_1444delinsC	p.Gly478Alafs*4	Frameshift	EGF7	1	0	Yes	Not present		
12	c.1452_1453del	p.Cys484*	Stop gain	EGF7	2	1	Yes	Not present		
12	c.1454delinsTGT	p.Glu485Valfs*2	Frameshift	EGF7	1	0	Yes	Not present		
12	c.1456dup	p.Arg486Lysfs*5	Frameshift		1	0	No	Not present	Hannoush, Puerta, Bauer, and Goldberg (2016)	
12	c.1459_1460del	p.Asp487Hisfs*3	Frameshift	EGF8	1	0	No	Not present	Crosnier et al. (1999)	
12	c.1461_1462del	p.Ile488Argfs*2	Frameshift	EGF8	1	0	No	Not Present	Krantz et al. (1998)	
12	c.1485_1486del	p.Cys496Phefs*9	Frameshift	EGF8	2	1	No	Not present	Crosnier et al. (1999); Bauer et al. (2010)	
12	c.1491del	p.Asn498Metfs*66	Frameshift	EGF8	1	0	Yes	Not present		
12	c.1499del	p.Gly500Valfs*64	Frameshift	EGF8	2	0	No	Not present	Warthen et al. (2006)	
12	c.1522dup	p.Arg508Lysfs*23	Frameshift	EGF8	1	0	No	Not present	Colliton et al. (2001)	
12	c.1526del	p.Phe509Serfs*55	Frameshift	EGF8	1	1	Yes	Not present		
12	c.1528 C>T	p.Gln510*	Nonsense	EGF8	2	2	No	Not present	Warthen et al. (2006)	
12	c.1538_1539del	p.Cys513Serfs*17	Frameshift	EGF8	1	0	No	Not Present	Colliton et al. (2001)	
12	c.1563_1564del	p.Cys522Serfs*8	Frameshift	EGF8	1	1	No	Not present	Warthen et al. (2006)	
12	c.1567 C>T	p.Gln523*	Nonsense	EGF8	1	0	No	Not present	Colliton et al. (2001)	
13	c.1641T>G	p.Tyr547*	Nonsense	EGF9	1	0	No	Not present	Colliton et al. (2001)	
13	c.1656del	p.Glu553Argfs*11	Frameshift	EGF9	1	2	No	Not present	Li et al. (1997)	
13	c.1657 G>T	p.Glu553*	Nonsense	EGF9	1	0	No	Not present	Colliton et al. (2001)	
13	c.1665T>G	p.Tyr555*	Nonsense	EGF9	1	0	No	Not present	Warthen et al. (2006)	
13	c.1678del	p.Cys560Alafs*4	Frameshift	EGF9	1	0	No	Not present	Lin et al. (2012)	
13	c.1682C>G	p.Ser561*	Nonsense	EGF9	1	0	Yes	Not present		
13	c.1713_1714del	p.Cys572*	Stop gain		1	0	No	Not present	Crosnier et al. (1999)	
13	c.1713dup	p.Cys572Leufs*2	Frameshift		1	1	No	Not present	Warthen et al. (2006)	
13	c.1713del	p.Cys572Valfs*3	Frameshift		1	0	No	Not present	Warthen et al. (2006)	
13	c.1720 G>C	Deletion of Exon 13	Splice site		1	0	No	Not present	Samejima et al. (2007)	Confirmed by cDNA
Intron 13	c.1720+2 T>C		Splice site		1	0	No	Not present	Warthen et al. (2006)	
14	c.1747del	p.Ala583Leufs*160	Frameshift	EGF10	1	0	No	Not present	Colliton et al. (2001)	
14	c.1802del	p.Pro601Leufs*142	Frameshift	EGF10	1	0	No	Not present	Colliton et al. (2001)	
14	c.1822C>T	p.Gln608*	Nonsense	EGF10	1	1	Yes	Not present		
14	c.1852_1858del	p.Asn618Alafs*123	Frameshift	EGF10	1	0	No	Not present	Colliton et al. (2001)	
14	c.1859dup	p.Phe621Leufs*8	Frameshift	EGF10	1	0	No	Not present	Krantz et al. (1998)	
14	c.1875C>G	p.Tyr625*	Nonsense	EGF10	1	0	No	Not present	Crosnier et al. (2000)	
15	c.1899_1900del	p.Cys633*	Stop gain	EGF11	2	1	No	Not present	Crosnier et al. (1999)	
15	c.1977 G>A	p.Trp659*	Nonsense	EGF11	1	0	No	Not present	Guo et al. (2018)	
15	c.1984del	p.Ala662Profs*81	Frameshift	EGF11	1	2	Yes	Not present		
15	c.1992T>A	p.Cys664*	Nonsense	EGF11	1	1	Yes	Not present		
16	c.2028 C>A	p.Cys676*	Nonsense	EGF12	1	0	Yes	Not present		
16	c.2039del	p.Gly680Alafs*63	Frameshift	EGF12	1	0	Yes	Not present		
16	c.2044dup	p.Cys682Leufs*7	Frameshift	EGF12	1	0	Yes	Not present		
16	c.2059_2060insT	p.Asn687Ilefs*2	Frameshift	EGF12	1	0	No	Not present	Krantz et al. (1998)	
16	c.2078 G>A	p.Cys693Tyr	Missense	EGF12	1	0	No	Not present	Warthen et al. (2006)	
16	c.2078_2079del	p.Cys693*	Stop gain	EGF12	1	0	Yes	Not present		
16	c.2084del	p.Asn695Metfs*48	Frameshift	EGF12	1	0	Yes	Not present		
16	c.2091 G>A	p.Trp697*	Nonsense	EGF12	1	0	No	Not present	Warthen et al. (2006)	
16	c.2096_2100del	p.Gly699Aspfs*6	Frameshift	EGF12	4	0	No	Not present	Krantz et al. (1998)	
17	c.2118_2119del	p.Asp706Glufs*4	Frameshift	EGF13	1	0	No	Not present	Krantz et al. (1998)	
17	c.2120dup	p.Ser707Argfs*4	Frameshift	EGF13	1	0	No	Not present	Colliton et al. (2001)	
17	c.2122 C>T	p.Gln708*	Nonsense	EGF13	1	0	No	Not present	Warthen et al. (2006)	
17	c.2122_2125del	p.Gln708Valfs*34	Frameshift	EGF13	9	3	No	Not present	Li et al. (1997)	
17	c.2141 G>A	p.Cys714Tyr	Missense	EGF13	1	2	No	Not present	Colliton et al. (2001); Tada et al. (2012)	
17	c.2173dup	p.Asp725Glyfs*4	Frameshift	EGF13	1	0	No	Not present	Krantz et al. (1998)	
17	c.2173del	p.Asp725Metfs*18	Frameshift	EGF13	1	0	Yes	Not present		
17	c.2204 G>A	p.Trp735*	Nonsense	EGF13	2	0	No	Not present	Pilia et al. (1999)	
17	c.2225_2226del	p.Ile742Serfs*5	Frameshift		2	0	No	Not present	Guegan et al. (2012)	
18	c.2230 C>T	p.Arg744*	Nonsense	EGF14	10	5	No	Not present	Krantz et al. (1998)	
18	c.2269_2270dup	p.Thr758Alafs*63	Frameshift	EGF14	1	0	No	Not present	Lin et al. (2012)	
18	c.2269_2270del	p.Gly757Hisfs*28	Frameshift	EGF14	1	0	Yes	Not present		
18	c.2276 G>T	p.Cys759Phe	Missense	EGF14	1	0	Yes	Not present		
18	c.2276delinsCA	p.Cys759Serfs*27	Frameshift	EGF14	1	1	Yes	Not present		
18	c.2279_2280del	p.Val760Glyfs*25	Frameshift	EGF14	1	0	No	Not present	Krantz et al. (1998)	
18	c.2304 C>A	p.Cys768*	Nonsense	EGF14	1	0	No	Not present	Jurkiewicz et al. (2005)	
Intron 18	c.2345–2 A>G		Splice site		1	1	No	Not present	Li et al. (2015)	
Intron 19	c.2372+1 G>T		Splice site		1	1	No	Not present	Kim, Yang, Paik, Choe, and Paik (2017)	
20	c.2418 C>A	p.Cys806*	Nonsense	EGF15	1	0	No	Not present	Colliton et al. (2001)	
20	c.2439_2442dup	p.Pro815Trpfs*10	Frameshift	EGF15	1	0	Yes	Not present		
20	c.2442dup	p.Pro815Alafs*9	Frameshift	EGF15	1	0	No	Not Present	Colliton et al. (2001)	
20	c.2455 A>G	Introduces Cryptic Splice Site	Splice site		1	1	Yes	Not present		Confirmed by cDNA
Intron 20	c.2458+1del		Splice site		1	0	No	Not present	Lin et al. (2012)	
Intron 20	c.2458+1 G>T		Splice site		1	0	No	Not present	Krantz et al. (1998)	
Intron 20	c.2458+5 G>A		Splice site		1	0	No	Not present	Warthen et al. (2006)	Unconfirmed by cDNA
Intron 20	c.2459–1 G>A		Splice site		1	1	No	Not present	Colliton et al. (2001)	
21	c.2473 C>T	p.Gln825*	Nonsense	EGF16	1	0	No	1/251,368 alleles	Krantz et al. (1998)	
21	c.2487 T>A	p.Cys829*	Nonsense	EGF16	1	0	Yes	Not present		
21	c.2505_2521del	p.Cys835Trpfs*38	Frameshift	EGF16	1	0	Yes	Not present		
21	c.2538 C>A	p.Cys846*	Nonsense	EGF16	1	0	No	Not present	Warthen et al. (2006)	
Intron 21	c.2572+1 G>A		Splice site		1	0	No	Not present	Warthen et al. (2006)	
Intron 21	c.2572+2 T>A	Deletion of Exon 22	Splice site		1	0	No	Not present	Warthen et al. (2006)	Confirmed by cDNA
22	c.2586_2589dup	p.Ile864Leufs*16	Frameshift	CRD	1	0	No	Not present	Warthen et al. (2006)	
22	c.2587dup	p.Cys863Leufs*16	Frameshift	CRD	1	0	No	Not present	Lin et al. (2012)	
22	c.2600_2601dup	p.Ser868Glyfs*3	Frameshift	CRD	1	0	No	Not present	Colliton et al. (2001)	
22	c.2601dup	p.Ser868Glufs*11	Frameshift	CRD	2	0	No	Not present	Oda et al. (1997)	
22	c.2606_2607del	p.Val869Aspfs*9	Frameshift	CRD	1	0	No	Not present	Krantz et al. (1998)	
22	c.2611_2612delinsTG	p.Pro871*	Stop gain	CRD	1	0	No	Not present	Krantz et al. (1998)	
22	c.2639_2640del	p.Cys880*	Stop gain	CRD	1	0	No	Not present	Oda et al. (1997)	
22	c.2662_2666delins21	p.Gly888Thrfs*69	Frameshift	CRD	1	1	No	Not present	Warthen et al. (2006)	Previously reported as c.2662_2677delins21
22	c.2666 G>A	p.Arg889Gln	Missense	CRD	2	1	No	17/282830 alleles	Warthen et al. (2006)	
22	c.2678 C>A	p.Ser893*	Nonsense	CRD	1	0	Yes	Not present		
22	c.2681dup	p.Val895Glyfs*57	Frameshift	CRD	1	0	No	Not present	Warthen et al. (2006)	
Intron 22	c.2682+1dup		Splice site		1	0	No	Not present	Warthen et al. (2006)	
Intron 22	c.2682+2 T>C		Splice site		1	0	No	Not present	Warthen et al. (2006)	
23	c.2688 G>A	p.Trp896*	Nonsense	CRD	2	0	No	Not present	Heritage et al. (2000)	
23	c.2691 T>A	p.Cys897*	Nonsense	CRD	1	0	No	Not present	Krantz et al. (1998)	
23	c.2698 C>T	p.Arg900*	Nonsense	CRD	5	0	No	Not present	Crosnier et al. (2000)	
23	c.2705 G>C	p.Cys902Ser	Missense	CRD	1	2	No	Not present	Colliton et al. (2001)	
23	c.2731dup	p.Cys911Leufs*41	Frameshift	CRD	1	0	Yes	Not present		
23	c.2732 G>A	p.Cys911Tyr	Missense	CRD	3	0	No	Not present	Warthen et al. (2006)	
23	c.2766_2773delinsTGCC	p.Asp923Alafs*21	Frameshift	CRD	1	0	Yes	Not present		
23	c.2770 C>T	p.Gln924*	Nonsense	CRD	2	0	No	Not present	Crosnier et al. (2000)	
23	c.2773_2774dup	p.Phe926Alafs*20	Frameshift	CRD	1	0	No	Not present	Colliton et al. (2001)	
23	c.2774_2788delinsCCAGGGCA	p.Cys925Serfs*18	Frameshift	CRD	1	1	No	Not present	Warthen et al. (2006)	Previously reported as c.2773_2787del15insCCAGGGCA
23	c.2798dup	p.Glu935Argfs*17	Frameshift	CRD	1	0	No	Not present	Warthen et al. (2006)	
23	c.2820_2826del	p.Leu941Argfs*2	Frameshift	CRD	1	0	No	Not present	Lin et al. (2012)	
23	c.2821_2831del	p.Leu941 Glufs*7	Frameshift	CRD	1	0	Yes	Not present		
23	c.2830dup	p.Val944Glyfs*8	Frameshift	CRD	1	0	No	Not present	Warthen et al. (2006)	
23	c.2844del	p.Cys948*	Stop gain	CRD	1	0	No	Not present	Warthen et al. (2006)	
23	c.2850del	p.Asp951Thrfs*19	Frameshift	CRD	1	0	No	Not present	Warthen et al. (2006)	
23	c.2874_2875del	p.Ala959Glufs*7	Frameshift	CRD	4	1	No	Not present	Crosnier et al. (2000)	
23	c.2898dup	p.Lys967Glnfs*16	Frameshift	CRD	1	0	No	Not present	Ropke et al. (2003)	
Intron 23	c.2916+1 G>A	Deletion of Exon 24	Splice site		1	0	No	Not present	Warthen et al. (2006)	Confirmed by cDNA
Intron 23	c.2916+1 G>C		Splice site		1	1	No	Not present	Oda et al. (1997)	
24	c.2918del	p.Gly973Valfs*11	Frameshift	CRD	1	0	Yes	Not present		
24	c.2923dup	p.Thr975Asnfs*8	Frameshift	CRD	1	2	No	Not present	Warthen et al. (2006)	
24	c.2927dup	p.Glu977Glyfs*6	Frameshift	CRD	1	0	Yes	Not present		
24	c.2935dup	p.Ile979Asnfs*4	Frameshift	CRD	1	0	No	Not present	Warthen et al. (2006)	
24	c.2956_2957dup	p.Leu986Phefs*2	Frameshift	CRD	1	0	Yes	Not present		
24	c.2960del	p.Asn987Ilefs*3	Frameshift	CRD	1	0	No	Not present	Colliton et al. (2001)	
24	c.2975_2978dup	p.Ala994Phefs*3	Frameshift	CRD	1	0	No	Not present	Warthen et al. (2006)	
24	c.2982_3000dup	p.Ala1001*	Stop gain	CRD	1	0	Yes	Not present		
24	c.2990 C>A	p.Ser997*	Nonsense	CRD	1	0	No	Not present	Li et al. (2015)	
24	c.3003_3008delinsGC	p.Cys1002Argfs*33	Frameshift	CRD	1	0	Yes	Not present		
24	c.3003_3006del	p.Cys1002Serfs*33	Frameshift	CRD	1	0	No	Not present	Colliton et al. (2001)	
24	c.3004 T>C	p.Cys1002Arg	Missense	CRD	1	0	Yes	Not present		
24	c.3011_3023dup	p.Asn1009Phefs*7	Frameshift		1	0	No	Not present	Li et al. (2015)	
24	c.3012_3013dup	p.Ser1005Phefs*32	Frameshift		1	0	No	Not present	Colliton et al. (2001)	
Intron 24	c.3048+1 G>T		Splice site		1	0	No	Not present	Boyer et al. (2005)	
25	c.3099del	p.Asp1033Glufs*3	Frameshift		1	0	No	Not present	Colliton et al. (2001)	
25	c.3103dup	p.Ile1035Asnfs*6	Frameshift		1	0	No	Not present	Warthen et al. (2006)	
25	c.3140 C>A	p.Ser1047*	Nonsense		1	0	No	Not present	Li et al. (2015)	
25	c.3160 G>T	p.Glu1054*	Nonsense		1	0	Yes	Not present		
25	c.3160_3163del	p.Glu1054*	Stop gain		1	0	No	Not present	Warthen et al. (2006)	
25	c.3163del	p.Val1055*	Stop gain		1	0	No	Not present	Warthen et al. (2006)	
25	c.3164_3167del	p.Val1055Glufs*7	Frameshift		3	0	No	Not present	Crosnier et al. (1999)	
25	c.3172 C>T	p.Gln1058*	Nonsense		2	0	Yes	Not present		
25	c.3197dup	p.Asp1067Argfs*42	Frameshift		1	0	No	Not present	Warthen et al. (2006)	
Intron 25	c.3200–2 A>G	Deletion of Exon 26	Splice site		1	0	No	Not present	Warthen et al. (2006)	Confirmed by cDNA
26	c.3203del	p.Phe1068Serfs*6	Frameshift	TM	1	0	No	Not present	Warthen et al. (2006)	
26	c.3218_3221dup	p.Ser1075Glyfs*35	Frameshift	TM	1	0	No	Not present	Giannakudis et al. (2001)	
26	c.3243dup	p.Ile1082Aspfs*27	Frameshift	TM	1	0	Yes	Not present		

Abbreviations: CRD, cysteine rich domain; DSL, delta/serate/lag‐2; EGF, epidermal growth factor; SP, signal peptide; TM, transmembrane.

*Note*: RefSeq NM_000214.2.

**Table 2:** *JAG1* pathogenic structural variants reported in our study

Mutation	Mutation type	Probands	Affected family members	Novel	Detection technique	References
Partial Exon 1 deletion (10673044–10673649)	Large deletion	1	1	Yes	Genome sequencing	Rajagopalan et al. *in preparation*
Deletion of exons 1 and 2 (c.5560_387+1662del)	Large deletion	1	2	No	MLPA	Warthen et al. (2006)
Deletion of exons 1 and 2	Large deletion	1	0	Yes	MLPA	
Deletion of exons 1 and 2	Large deletion	1	0	Yes	MLPA	
Deletion of exons 1–23	Large deletion	1	0	Yes	MLPA	
Deletion of exon 3	Large deletion	1	0	Yes	Sequencing cDNA	
Deletion of exon 3	Large Deletion	1	1	Yes	MLPA	
Deletion of exons 3–26	Large deletion	1	0	Yes	MLPA	
Deletion of exons 9–12	Large deletion	1	0	No	MLPA	Lin et al. (2012)
Deletion of exons 10–26	Large deletion	1	0	Yes	MLPA	
Deletion of exons 22–24	Large deletion	1	0	Yes	MLPA	
257 Kb Deletion (10402074–10658979)	Large deletion	1	0	Yes	SNP array	
0.095 Mb Deletion (10508458–10604087)	Large deletion	1	0	No	SNP array	Kamath et al. (2009)
0.85 Mb Deletion (9867383–10717877)	Large deletion	1	0	No	SNP array	Laufer‐Cahana et al. (2002); Kamath et al. (2009)
861 Kb Deletion (10523952–11385119)	Large deletion	1	0	Yes	SNP array	
1.18 Mb Deletion (9510922–10691024)	Large deletion	1	0	No	SNP array	Kamath et al. (2009)
1.55 Mb Deletion (9473601–11027205)	Large deletion	1	0	No	SNP array	Kamath et al. (2009)
2.44 Mb Deletion (10308051–12746054)	Large deletion	1	0	No	SNP array	Kamath et al. (2009)
2.83 Mb Deletion 9452468–12280526	Large deletion	1	0	No	SNP array	Kamath et al. (2009)
2.84 Mb Deletion (8647519–11490289)	Large deletion	1	0	No	SNP array	Lin et al. (2012)
2.96 Mb Deletion (9251107–12214763)	Large deletion	1	0	No	SNP array	Lin et al. (2012)
3.03 Mb Deletion (8813006–11844206)	Large deletion	1	0	No	SNP array	Warthen et al. (2006); Kamath et al. (2009)
3.28 Mb Deletion (10380766–13660393)	Large deletion	1	0	No	SNP array	Kamath et al. (2009)
3.84 Mb Deletion (8521437–12366350)	Large deletion	1	0	No	SNP array	Kamath et al. (2009)
3.89 Mb Deletion (7383615–11268829)	Large deletion	1	0	No	SNP array	Kamath et al. (2009)
4.00 Mb Deletion 8044130–12044897	Large deletion	1	0	No	SNP array	Kamath et al. (2009)
4.17 Mb Deletion (9952874–14120443)	Large deletion	1	0	No	SNP array	Kamath et al. (2009)
4.44 Mb Deletion (8756310–13195568)	Large dDeletion	1	0	No	SNP array	Kamath et al. (2009)
5.66 Mb Deletion (7054353–12717237)	Large deletion	1	0	No	SNP array	Kamath et al. (2009)
5.97 Mb Deletion (9039235–15011679)	Large deletion	1	0	No	SNP array	Kamath et al. (2009)
7.40 Mb Deletion (3987627–11390419)	Large deletion	1	0	No	SNP array	Kamath et al. (2009)
8.31 Mb Deletion (5709024–14014276)	Large deletion	1	0	No	SNP array	Kamath et al. (2009)
10.57 Mb Deletion (9685413–20253840)	Large deletion	1	0	Yes	SNP array	
11.96 Mb Deletion (14300641–26257255)	Large deletion	1	0	No	SNP array	Kamath et al. (2009)
Gene deletion	Large deletion	1	0	No	FISH	Rand, Spinner, Piccoli, Whitington, and Taub (1995)
Gene deletion	Large deletion	1	0	Yes	MLPA	
Gene deletion	Large deletion	1	0	Yes	MLPA	
Gene deletion	Large deletion	1	0	Yes	MLPA	
Gene deletion	Large deletion	1	0	Yes	MLPA	
Duplication of exons 1–25	Large duplication	1	0	Yes	MLPA	
Duplication of exons 3 and 4	Large duplication	1	0	No	MLPA	Warthen et al. (2006)
Inversion (10663195–11342633)	Inversion	1	1	Yes	Genome sequencing	Rajagopalan et al. in preparation
t(2;20)(q21.3;p12)	Translocation	1	1	No	FISH/Karyotype	Spinner et al. (1994); Kamath et al. (2009)
t(1;20)(p.22.1;p11.2)	Translocation	1	0	No	FISH/Karyotype	Warthen et al. (2006)

Abbreviations: FISH, flourescence in situ hybridization; MLPA, multiplex ligation‐dependent probe amplification; SNP, single nucleotide polymorphism.

*Note*: RefSeq NM_000214.2.

Our data show that the most common mutation types are frameshift (37%), followed by nonsense (22%), large gene deletions (13%), missense (13%), splice site (12%), in‐frame deletions (1%), partial gene duplications (<1%), translocations (<1%), inversions (<1%), and start loss variants (<1%; Figure S2).

**Figure 2:**
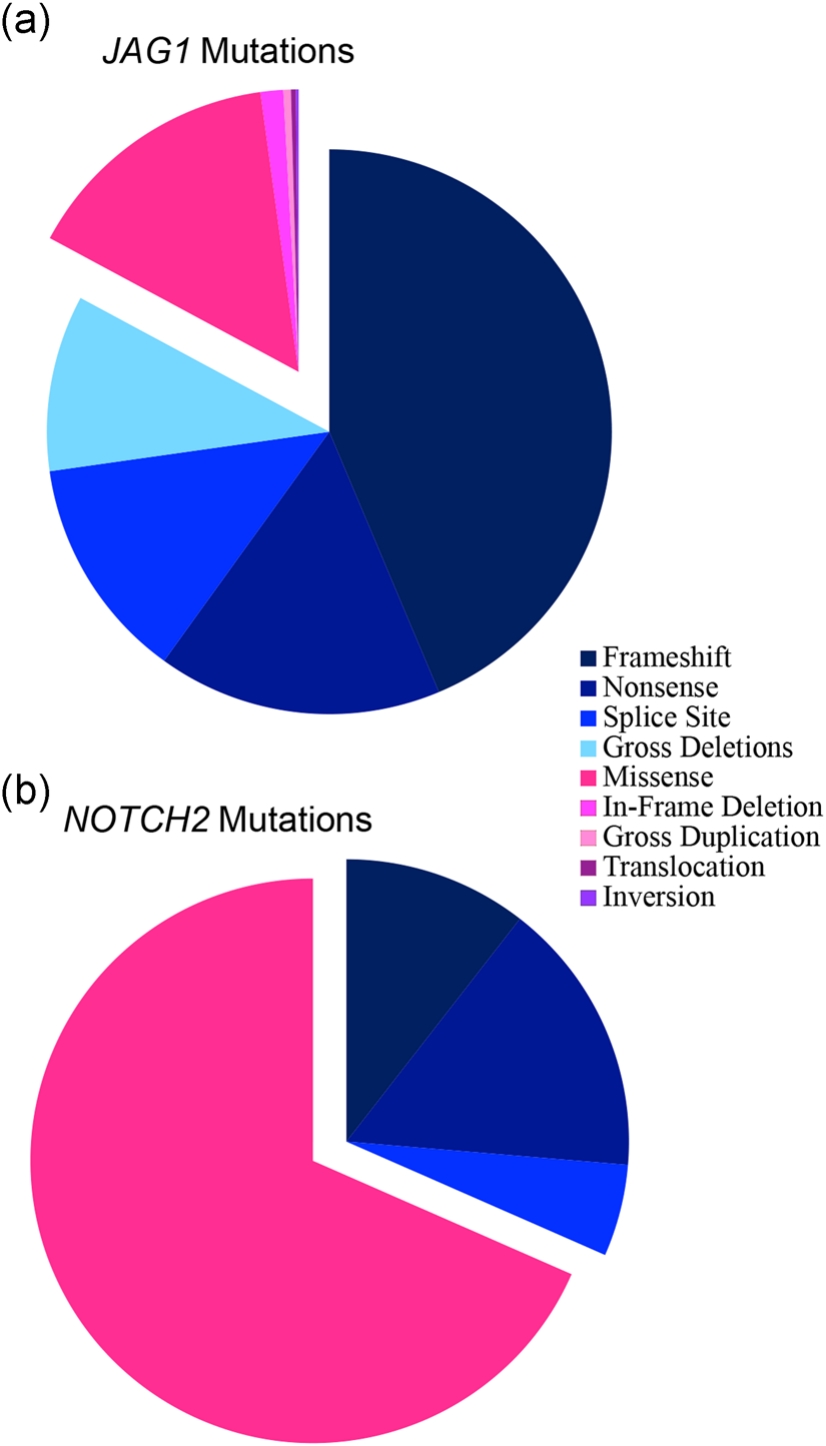
Incidence of all reported and novel *JAG1* and *NOTCH2* mutation types. (a) *JAG1* protein‐truncating pathogenic variants are shown in blue color tones and include: frameshift (*n* = 303), nonsense (*n* = 113), splice site (*n* = 89), and gross deletion (*n* = 70). *JAG1* non‐protein‐truncating pathogenic variants are shown in pink color tones and include: missense (*n* = 104), in‐frame deletion (*n* = 9), gross duplication (*n* = 3), translocation (*n* = 2), and inversion (*n* = 1). (b) *NOTCH2* protein‐truncating pathogenic variants are shown in blue color tones and include: frameshift (*n* = 2), nonsense (*n* = 3), and splice site (*n* = 1). *NOTCH2* non‐protein‐truncating pathogenic variants are shown in pink color tones and include: missense (*n* = 13)

Frameshift variants are predominantly caused by deletions (51%) and duplications (40%), but are also caused by insertions–deletions (indels, 8%), and rarely by insertions (1%). Seventy‐eight percent of nonsense variants are caused by single nucleotide substitutions. Stop gain variants account for 20% of nonsense variants, and occur through deletions (77%), indels (15%), and duplications (8%). A single start loss variant accounts for the remaining 2% of nonsense variants. The overwhelming majority of splice site variants are due to single nucleotide substitutions (83%), with the remaining 17% caused by deletions, duplications, or indels at or near the splice site. The incidence of each mutation type is relatively unchanged when our data set is combined with all reported pathogenic and likely pathogenic variants (totaling 694 unique variants), and has also remained relatively stable in the 27 years because pathogenic variants in *JAG1* were first identified as the cause of ALGS, suggesting that these frequencies are an accurate indication of mutation‐type prevalence in *JAG1* for ALGS (Crosnier et al., 1999; Stenson et al., 2017; Warthen et al., 2006; Figure 2a).

### 3.2 Large gene deletions

Large gene deletions differ in both length and in the location of their breakpoints, two findings that have previously been used to suggest that there is no specific genomic hotspot for rearrangement. It has been recognized that patients with 20p deletions can have other abnormalities, including developmental delay, hearing loss, and autism, among others, and work by Kamath et al. defined a 5.4 Mb region, including 12 genes, within which deletions led to ALGS‐specific disease phenotypes (Kamath et al., 2009). They further showed that individuals with deletion variants extending distally or proximally from this region all presented with additional phenotypes. Of the 44 deletions that we report here, we provide mapped breakpoints for 23 (52%) of them, of which 3 have not previously been described. These three deletions include two that fall within the 5.4 Mb ALGS‐specific region (257 and 861 Kb) and one that is larger (10.57 Mb). Clinical data from the two patients with the smaller deletions does not include phenotypes outside of ALGS; however, we only have records from infancy and we cannot speculate whether additional conditions arose with age. Clinical data from the patient with the 10.57 Mb deletion includes obesity and significant developmental delay.

### 3.3 JAG1 missense variants

**Figure 3:**
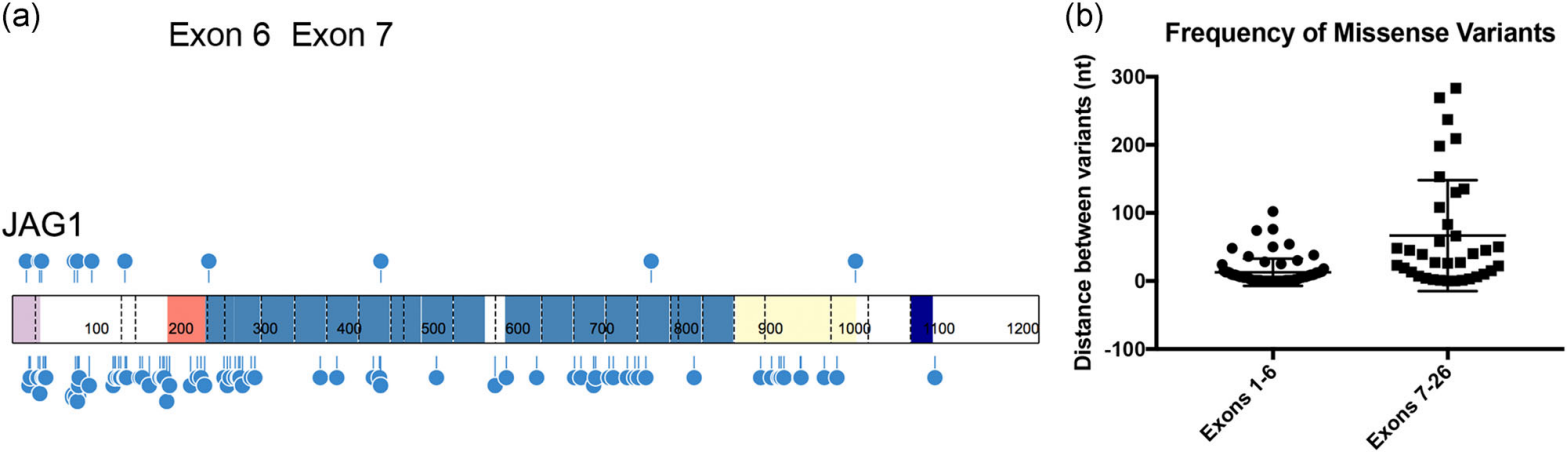
JAG1 missense variants cluster in the N‐terminus. (a) Schematic showing all reported (lower) and novel (upper) missense mutations in JAG1. Dashed lines within the protein indicate exon boundaries and numbers indicate amino acid coordinates. (b) Distance in nucleotides between missense mutations within exons 1–6 and exons 7–26. Statistical significance (*p* < .0001) was calculated using an unpaired, two‐tailed *t* test. RefSeq NM_000214.2. Protein schematic was prepared using ProteinPaint software from Saint Jude Children's Research Hospital–Pediatric Cancer Data Portal (Zhou et al., 2016)

**Figure 4:**
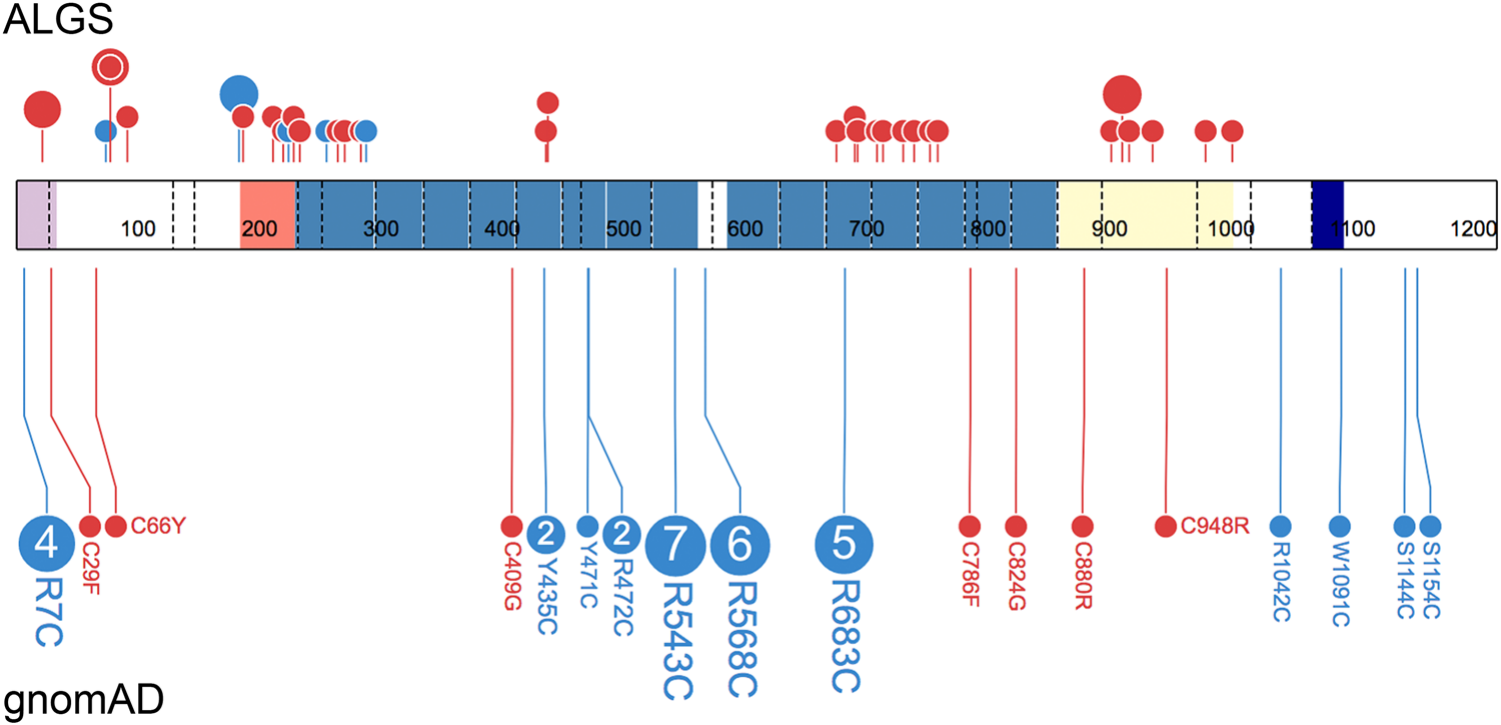
Gain of cysteine missense variants are more tolerated in JAG1. Schematic showing all missense variants involving gain (blue) or loss (red) of a cysteine in JAG1 from control samples present in gnomAD (lower) and in patients with ALGS (upper). The disease population includes combined data from HGMD, ClinVar, LOVD, and novel mutations reported here. Numbers within the circle indicate the number of alleles seen for each variant in gnomAD. Circle size and height is proportional to the number of probands with that variant in the disease population. The concentric circle in the ALGS cohort indicates multiple variants at the same amino acid position (p.C78Y, p.C78G, p.C78R, and p.C78S). RefSeq NM_000214.2. Protein schematic was prepared using ProteinPaint software from Saint Jude Children's Research Hospital–Pediatric Cancer Data Portal (Zhou et al., 2016)

Missense variants were found throughout the entire extracellular region of the gene, with a statistically significant overrepresentation (*p* = .0002; unpaired, two‐tailed *t* test) of missense variants clustering within the first 6 exons of the gene, an observation that has previously been reported (Masek & Andersson, 2017; Spinner et al., 2001; Figure S3). The statistical significance increases (*p* < .0001; unpaired, two‐tailed *t* test) when reported pathogenic or likely pathogenic missense variants that are not present in our cohort are added to our data set (Figure 3). Overall, 15% of all *JAG1* pathogenic or likely pathogenic variants (our cohort and previously reported variants, *n* = 104 of 694) are missense. Almost a quarter of these *JAG1* missense variants involve the gain or loss of a cysteine within the EGF‐like domain (*n* = 22 of 104, 21% of total reported and novel variants). The importance of cysteine in the proper folding of the EGF‐like domain in both ALGS as well as other syndromes, including cerebral autosomal dominant arteriopathy with subcortical infarcts and leukoencephalopathy (CADASIL) and Marfan syndrome, has previously been described, and it is accepted that variants of this kind in this region are very likely disease‐causing (Bauer et al., 2010; Haritunians et al., 2005; Le Caignec et al., 2002; Schrijver, Liu, Brenn, Furthmayr, & Francke, 1999; Whiteman et al., 2007). To further understand cysteine changes in relation to disease, we plotted the frequency and distribution of cysteine changes observed in gnomAD compared to all cysteine changes reported in ALGS (including our cohort), and found that cysteine loss was more prevalent in the disease population whereas cysteine gain was overrepresented in the control population derived from gnomAD, suggesting a greater tolerance for cysteine gain in healthy individuals (Figure 4). We also observed an inverse correlation in clusters of reported pathogenic or likely pathogenic variants found in the disease population compared to clusters of variants reported in gnomAD, highlighting possible hotspots for pathogenic cysteine variant occurrence as well as genomic regions that appear more tolerant to cysteine changes.

**Figure 5:**
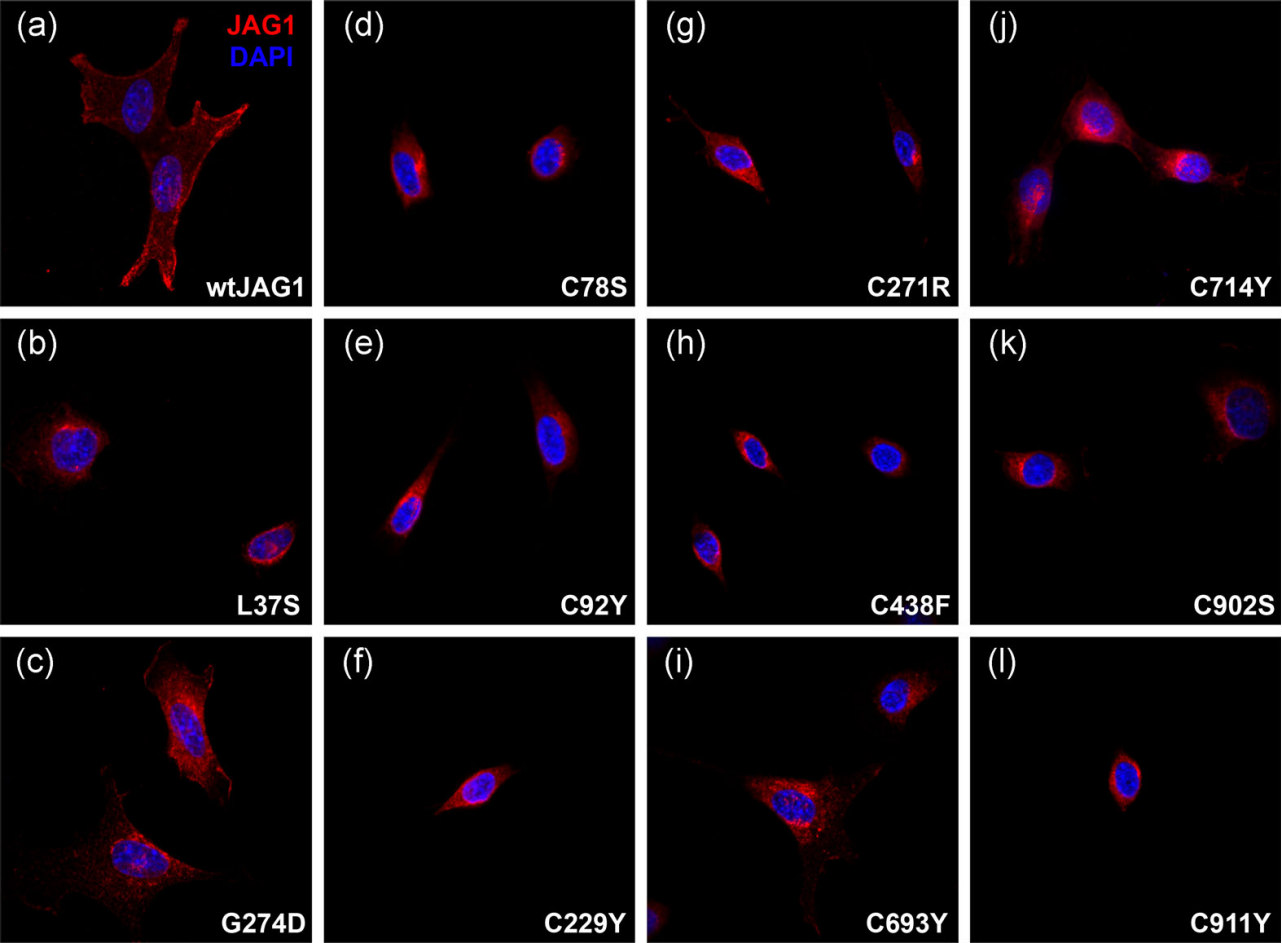
Cysteine‐loss missense variants are defective in protein localization. Confocal microscopy of stably‐transfected NIH‐3T3 cells expressing the following controls: (a) wild type JAG1 and two positive controls with known nuclear retention and perinuclear localization (b) p.G274D and (c) p.L37S (Lu et al., 2003; Morrissette et al., 2001). (d–l) Cysteine‐loss missense variants all show protein clustering near the nuclei

**Figure 6:**
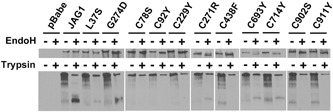
Cysteine‐loss missense variants are differentially sensitive to enzymatic proteolysis. Western blot of protein lysates from stably transfected NIH‐3T3 cells treated with Endo H or Trypsin. Controls include wild type JAG1 (not sensitive), p.L37S (sensitive), and p.G274D (partially sensitive)

Given the propensity for cysteine‐loss in the disease population, we studied the functional effect of nine cysteine‐loss missense variants by assaying for cellular localization, glycosylation, and Notch signaling ability. Wild type JAG1 is normally expressed on the cell surface, where it can interact with Notch receptors and activate Notch signaling. Immunofluorescence of stable cell lines showed perinuclear retention of six different JAG1 variants (p.Cys78Ser, p.Cys92Tyr, p.Cys229Tyr, p.Cys438Phe, p.Cys902Ser, and p.Cys911Tyr), indicating that these mutant JAG1 proteins are not properly localized (Figure 5). The remaining three variants (p.Cys271Arg, p.Cys693Tyr, and p.Cys714Tyr) showed weak expression of JAG1 on the cell membrane as well as perinuclear retention, indicating a partial defect in protein localization. To complement these data, we treated cells expressing each JAG1 missense variant with trypsin, which degrades proteins present on the cell surface, but will not degrade proteins that are trapped intracellularly. Again, the same six missense variants found to show complete perinuclear retention (p.Cys78Ser, p.Cys92Tyr, p.Cys229Tyr, p.Cys438Phe, p.Cys902Ser, and p.Cys911Tyr) were similarly protected from proteolysis by trypsin, indicating that they were not present on the cell surface, whereas the three missense variants with weak cell surface expression (p.Cys271Arg, p.Cys693Tyr, and p.Cys714Tyr) were likewise partially susceptible to proteolysis (Figure 6).

Wild‐type JAG1 harbors complex‐type N‐glycans that are unaffected by endoglycosidase H (Endo H), an enzyme that cleaves high‐mannose and hybrid‐type N‐glycans, but not complex‐type N‐glycans (Bauer et al., 2010; Freeze & Kranz, 2010; Morrissette et al., 2001). Sensitivity of glycoproteins to Endo H that are normally resistant, like JAG1, typically indicates that the protein is trapped in the secretory pathway, likely sequestered in either the endoplasmic reticulum or the cis Golgi (Freeze & Kranz, 2010). Western blots from all nine missense variants indicate the presence of a smaller molecular weight protein after treatment with Endo H, whereas the molecular weight of the wild‐type JAG1 is unaltered, indicating improper posttranslational glycosylation of all nine missense variants (Figure 6). As with the trypsin assay, three of the missense variants (p.Cys271Arg, p.Cys693Tyr, and p.Cys714Tyr) displayed partial cleavage, indicating that these JAG1 proteins are partially trapped in the secretory pathway.

**Figure 7:**
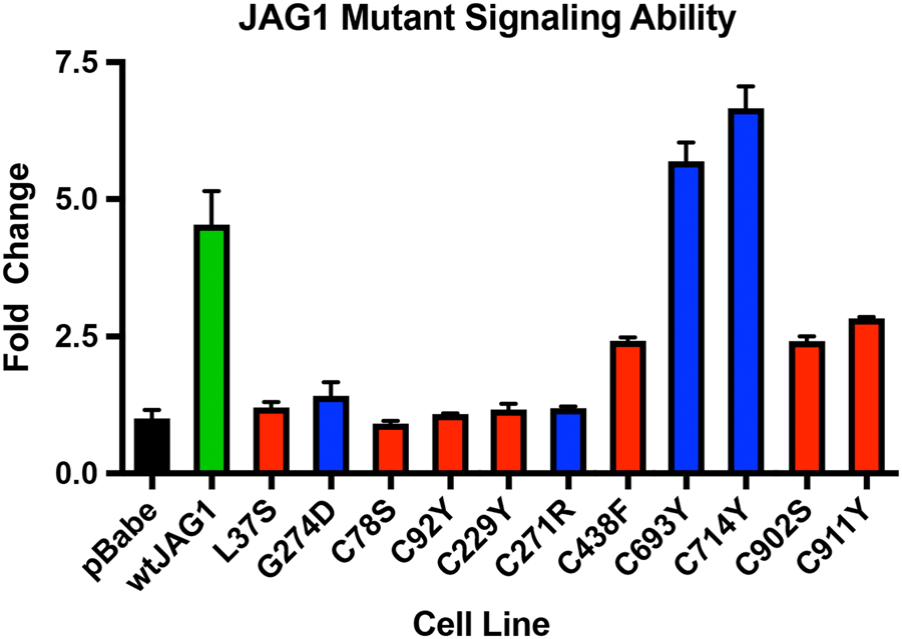
Luciferase assay of cysteine‐loss missense variants showing reduced JAG1 signaling. Luciferase assay of NIH‐3T3 cells transfected with 4XCBF‐luciferase reporter construct and cocultured with wild type or mutant JAG1‐expressing cells. RLU signals were normalized to internal *Renilla* controls. p.L37S and p.G274D are included as negative controls. All variants showed a statistically significant decrease in luciferase activity (unpaired, two‐tailed *t* test) when compared to wild type, with the exception of p.C693Y, which was statistically unchanged from wild type, and p.C714Y, which showed a statistically significant increase in luciferase activity from wild type. Red bars indicate variants that are not expressed on the cell membrane and blue bars indicate variants that have some partial expression on the cell membrane (based on results from Figures 5 and 6)

Using a luciferase assay, we further tested the ability of each mutant protein to activate Notch signaling by exposure to a reporter construct containing four tandem Notch‐responsive CBF binding sites in the promoter region of the luciferase gene (Hsieh et al., 1996). Here, we found that seven of the nine mutants were unable to increase luciferase activity (p.Cys78Ser, p.Cys92Tyr, p.Cys229Tyr, C271R, p.Cys438Phe, p.Cys902Ser, and p.Cys911Tyr) whereas two retained Notch signaling function (p.C693Y and p.C714Y; Figure 7).

The majority of the variants we tested are defective in all three categories (trafficking, glycosylation, and Notch signaling ability), and are truly null alleles. Enzymatic data from three variants suggests that they are partially trapped within the secretory pathway in agreement with immunofluorescence staining showing defective trafficking (p.Cys271Arg, p.Cys693Tyr, and p.Cys714Tyr). Of those variants, p.C271R is unable to activate Notch signaling, and therefore cannot propagate a signal. The other two variants, p.C693Y and p.C714Y, are able to activate Notch signaling to a similar degree as wild type JAG1, and therefore impaired signaling is not the molecular basis for the ALGS phenotypes seen in individuals with these mutations. Our other assays do show a partial defect in cellular localization, although some protein is still expressed on the cell surface where it could theoretically participate in Notch signaling. Both probands with the p.C693Y and p.C714Y variants in our cohort, however, do not have features that would distinguish them from other individuals with ALGS, and their overlapping symptoms include bile duct paucity, peripheral pulmonic stenosis, and facies, with cholestasis, liver transplant, heart murmur, and posterior embryotoxon also present in the individual with the c.C714Y variant. Variants have previously been described to be “leaky,” meaning that proteins retain partial, albeit reduced, wild type function, and indeed we included the known pathogenic variant p.G274D as a positive control, which has been shown to have impaired signaling ability but only a partial loss in cellular localization/trafficking (Bauer et al., 2010; Lu, Morrissette, & Spinner, 2003; Morrissette et al., 2001). Efforts to identify whether variants that retain some partial protein function lead to milder or cardiac‐specific clinical features have proven inconclusive, but suggest that there may be a threshold for JAG1 haploinsufficiency (Bauer, 2010). It is also possible that there are innate cellular differences between the in vitro signaling assay and the in vivo environment of the developing liver. Vascular smooth muscle cells, which express NOTCH3 (Baeten & Lilly, 2017), are also likely to be a major source of JAG1 during biliary development, and it is possible that these mutations (p.C693Y and p.C714Y) enhance binding of JAG1 and NOTCH3, thus, reducing JAG1 function through NOTCH2 in vivo. A functional understanding of how these two variants result in ALGS phenotypes that are indiscriminate from those caused by other mutations suggests that biological relevance may be a possible limitation of the in vitro signaling assay. These data highlight a heterogeneity in the functional consequences of pathogenic ALGS variants.

## 4 VARIANTS IN *NOTCH2*

We identified nine unique *NOTCH2* variants in 10 of 401 (2.5%) probands in our cohort. These variants are predominantly missense, but also include splice site and nonsense variants (Table 3; Figure S4). Three of these pathogenic *NOTCH2* variants have not previously been described, which brings the total number of known pathogenic *NOTCH2* variants to 19, and we describe the clinical features of the individuals with these novel variants in Table S1. All three of the reported nonsense variants cluster within the intracellular domain, with two occurring within the ANK repeats. Pathogenic variants have not been identified in every exon, and it is unknown whether this, or the intracellular localization of nonsense variants, is due to mutation hotspots in the gene or the small sample size of affected people with confirmed pathogenic *NOTCH2* variants.

**Table 3:** *NOTCH2* pathogenic variants reported in our study

Exon	DNA variant	Protein change	Coding effect	Protein domain	Probands	Affected family members	Novel	Frequency in gnomAD	References
7	c.1117 T>C	p.Cys373Arg	Missense	EGF9	1	4	No	Not present	Kamath et al. (2012)
7	c.1147 C>T	p.Pro383Ser	Missense	EGF10	1	1	No	Not present	Kamath et al. (2012)
8	c.1331 G>A	p.Cys444Tyr	Missense	EGF11	1	2	No	Not present	McDaniell et al. (2006)
8	c.1418 A>G	p.Asp473Gly	Missense	EGF12	1	1	Yes	Not present	
8	c.1418 A>T	p.Asp473Val	Missense	EGF12	1	1	Yes	Not present	
27	c.4967 A>G	p.Gln1656Arg	Missense		1	0	Yes	16/251474 Alleles	
32	c.5857 C>T	p.Arg1953Cys	Missense	ANK4	1	0	No	Not present	Kamath et al. (2012)
Intron 32	c.5930–1 G>A		Splice site		1	1	No	Not present	McDaniell et al. (2006)
33	c.6007 C>T	p.Arg2003*	Nonsense	ANK6	2	0	No	Not present	Kamath et al. (2012)

*Note*: RefSeq NM_024408.3.

When combined with reported data, missense variants remain the most common mutation type for *NOTCH2* (*n* = 13 out of 19, 68%; Figure 1b and Figure 2b; Kamath et al., 2012; Liu, Wang, Dong, Feng, & Huang, 2018; McDaniell et al., 2006). A majority of missense variants are found in exons 7 and 8 (*n* = 7 of 13; 54%) and an additional four missense variants occur in exons 31 and 32 (31%), indicating that screening of these 4 exons alone captures 85% of reported pathogenic or likely pathogenic variants.

## 5 MUTATION NEGATIVE PROBANDS

Combined sequencing of *JAG1* and *NOTCH2* along with copy number variant analysis of *JAG1* by MLPA did not result in pathogenic variant identification in 13 out of 401 (3.2%) probands in our cohort, despite this group of people meeting the diagnostic criteria for ALGS (Table S2).

## 6 CLINICAL RELEVANCE

### 6.1 Classification of JAG1 missense variants

*JAG1* variants that result in a truncated or absent protein comprise the largest group of reported disease‐causing variants (83%). Given the proposed haploinsufficient nature of the disease, these variants are very likely to be disease‐causing. There is more of a need, however, to confirm the pathogenicity of missense variants, both in *JAG1* (*n* = 104 of 694; 15%) and in *NOTCH2* (*n* = 13 of 19; 68%). We have shown here that *JAG1* missense variants involving the gain of a cysteine appear to be more tolerated, as they are overrepresented in the general population, whereas those involving the loss of a cysteine are more commonly associated with disease. Ultimately, we hope that these observations are able to better guide missense variant interpretation in ALGS.

Functional characterization is necessary to conclusively classify missense variants, and our group and others have shown that many pathogenic missense variants result in improper protein folding, incorrect cellular localization, and/or a defect in Notch signaling activation (Bauer et al., 2010; Guarnaccia, Dhir, Pintar, & Pongor, 2009; Lu et al., 2003; Morrissette et al., 2001; Tada, Itoh, Ishii‐Watabe, Suzuki, & Kawasaki, 2012). However, these studies have also categorized variants that were thought to be disease‐causing as benign, which highlights the need for functionally validating individual variants (Bauer et al., 2010; Morrissette et al., 2001; Tada et al., 2012). Interestingly, while we and others had previously proposed that JAG1 variants that are not wholly defective in both Notch signaling ability and proper cellular localization might confer a milder, non‐ALGS phenotype, we show here for the first time that these variants are present in patients with full features of ALGS.

The majority of *JAG1* missense variants do not result in the gain or loss of a cysteine and/or are not found within one of the EGF‐like domains (82 out of 104, 78% of reported and novel variants). Many of these variants are found within the first six exons of the *JAG1* gene, which we show contains a statistically significant greater number of missense variants than exons 7–26 (Masek & Andersson, 2017; Spinner et al., 2001). These first six exons encode the signal peptide, the DSL domain, and the first two EGF‐like domains of the JAG1 protein. The DSL domain is required for effective binding to NOTCH2, whereas the signal peptide is necessary for proper trafficking of the mature protein to the cell membrane (Kopan & Ilagan, 2009; Lindsell et al., 1995). The finding that these regions contain a hotspot for pathogenic missense variants suggests that these functional motifs are particularly susceptible to single nucleotide changes. However, a recent study in mice aimed to analyze the missense variant H268Q, which occurs in the homologous hotspot region in mice, surprisingly showed that the mature Jag1 protein was able to interact and signal through Notch2 although still resulting in eye, heart, and liver defects similar to ALGS (Andersson et al., 2018; Hansson et al., 2010). These results offer insight into how pathogenic missense variants in this region may affect JAG1 protein function and ultimately result in ALGS and highlight the importance of functionally validating individual variants to confirm whether the same type of physiological consequence is observed.

We have combined our data with sequence classification criteria outlined by the American College of Medical Genetics (ACMG) to categorize all nine missense variants for which we provide functional data in this report, as pathogenic or likely pathogenic (Richards et al., 2015; Table S3). Along with these nine cysteine missense variants, we have additionally reviewed the remaining 29 missense variants that we report in our cohort and have classified 32 as pathogenic or likely pathogenic and six as variants of uncertain significances (VUSs) (Table S3). The majority of these variants have been previously reported, many with functional data, which supports their pathogenicity. However, 6 of 38 variants (16%) have limited evidence to support disease causality. In most instances, the identification of more individuals with ALGS who have these variants or functional validation will be enough to elevate their classification to likely pathogenic. One variant reported here is present at a frequency of 17/282830 alleles (p.Arg889Gln), and we classified this as a VUS.

### 6.2 Classification of NOTCH2 missense variants

Unlike *JAG1*, pathogenic variants in *NOTCH2* are predominantly missense (13 of 19, 68% reported and novel variants). We observed two hubs for increased pathogenic missense variant frequency in the *NOTCH2* gene, which together account for 85% of reported missense variants. The first hub occurs in exons 7 and 8, which alone harbor 54% of reported pathogenic missense variants, of which we see five in our cohort. These two exons encode EGF‐like domains (EGF repeats 9–12) of *NOTCH2*. The second hub occurs in exons 31 and 32, which accounts for 31% of reported pathogenic missense variants, of which we see four in our cohort. These two exons code for the Ankyrin (ANK) repeat domain of *NOTCH2*. A few of these missense variants have been studied to determine their functional consequence by assaying their ability to be activated by JAG1 using luciferase reporters, which confirmed pathogenicity in five out of six tested variants (Kamath et al., 2012). Little else has been done to specifically interrogate *NOTCH2* missense variants in the context of ALGS, however a study in fruit flies found that a specific missense variant, V361M, located within an EGF‐like domain was able to discriminate between ligands, such that it effectively abrogated the ability of Serrate (Jagged homolog) ligands to signal through NOTCH, whereas Delta (Delta‐like homolog) ligands were able to signal normally, thus defining a domain that specifically affects Serrate‐binding (Yamamoto et al., 2012). Additional work in NOTCH1 has identified a minimal region of EGF repeats (EGF repeats 6–15) that are sufficient to fully activate signaling in an in vitro reporter assay (Andrawes et al., 2013), and this combined with work by Yamamoto et al. (2012) supports a growing hypothesis that missense variants within this region are less tolerated and more likely to confer a functional consequence. It will be interesting to see if some of the identified missense variants in ALGS act similarly.

## 7 DIAGNOSTIC RELEVANCE AND FUTURE PROSPECTS

Results from our comprehensive 27‐year, single‐center study provides updated statistics regarding the incidence of *JAG1* (94.3%; *n* = 377 out of 401), *NOTCH2* (2.5%; *n* = 10 of 401), and mutation negative cases (3.2%; *n* = 13 of 401) of ALGS. In addition, we report 86 novel *JAG1* pathogenic variants and three novel *NOTCH2* pathogenic variants. When combined with previously published data, we provide the most up‐to‐date data on the frequency of mutation‐type seen in patients with *JAG1* or *NOTCH2* pathogenic variants.

Successful screening of patients necessitates both sequencing and copy number analysis, which can be carried out by Sanger sequencing and MLPA, or next generation sequencing (NGS) with copy number variation analysis across the gene (Gilbert, 2018; Spinner, Leonard, & Krantz, 2013). The current standard is to sequence all exons in *JAG1*, which should identify approximately 85% of ALGS pathogenic variants. If CNV analysis is not carried out simultaneously with sequencing, second tier diagnostics involves large deletion/duplication analysis through either multiplex ligation‐dependent probe amplification (MLPA), chromosomal microarray (CMA), or fluorescence in situ hybridization (FISH), which should identify an additional 9% of pathogenic variants. Samples without an identified *JAG1* pathogenic variant would then undergo Sanger sequencing for *NOTCH2*, which should uncover an additional 2–3% of pathogenic variants.

A notable finding from our study is the percentage (3.2%) of mutation negative individuals that we describe. These individuals have all met the standards for clinical classification of ALGS, but do not have a pathogenic variant in *JAG1* or *NOTCH2*. We hypothesize that these include patients with *JAG1* variants not previously identified by conventional testing (Sanger sequencing and MLPA), as well as a subset of patients that will be found to have a different diagnosis with overlapping features of Alagille syndrome. The best approach towards a molecular understanding of this population is to perform more comprehensive sequencing methodologies, including whole exome sequencing (ES), whole genome sequencing (GS), and/or RNA sequencing (RNAseq). Using ES, we have previously identified compound heterozygous pathogenic variants in the gene *ATP8B1*, a gene involved in progressive familial intrahepatic cholestasis type I (PFIC1) in a patient with overlapping features of ALGS and PFIC1 (Grochowski et al., 2015). Individuals with ABCB4 deficiency, which results in a variety of hepatic phenotypes including PFIC Type 3, have also been misdiagnosed as having ALGS (Schatz et al., 2018). Similarly, siblings with an initial diagnosis of ALGS were found to have a pathogenic variant in the *NEK8* gene, which is commonly mutated in renal‐hepatic‐pancreatic‐dysplasia 2 (RHPD2) and in nephronophthisis (NPHP9), and resulted in a reclassification of the disease to encompass a spectrum of disorders that involve *NEK8* pathogenic variants rather than ALGS (Rajagopalan et al., 2016). These studies suggest that full evaluation of our 13 mutation negative individuals, which has not yet been performed, may lead to disease reclassification.

Given the obvious molecular etiology of ALGS as a disease of Notch signaling dysfunction, we anticipate that regulatory regions within *JAG1* or *NOTCH2*, or regions within those two genes that are missed by more traditional sequencing technologies, including ES, are the most likely candidates for novel molecular discovery. The advanced technology provided by GS is able to identify more complicated structural variants in *JAG1*, and indeed we describe here a partial gene deletion and an inversion detected by GS (Rajagopalan et al., in preparation). We are confident that a larger subset of mutation negative individuals with a clear clinical indication of ALGS will be definitively diagnosed as we screen this cohort.

## 8 CONCLUSIONS

Overall, our decades‐long study on ALGS has allowed us to accumulate comprehensive information on the types and frequencies of mutations in ALGS. We report an additional 86 *JAG1* pathogenic variants and three *NOTCH2* pathogenic variants, bringing the total number of described variants to 694 and 19, respectively (Stenson et al., 2017). We find that 94.3% of individuals with clinically diagnosed ALGS have a pathogenic variant in the *JAG1* gene, 2.5% have a pathogenic variant in the *NOTCH2* gene, and 3.2% are molecularly uncharacterized. We caution other researchers and clinicians on the functional relevance of missense variants, both in *JAG1* and particularly in *NOTCH2*, where they predominate. Finally, we suggest that NGS strategies may best interrogate the small population of molecularly undiagnosed patients, and that these approaches should prioritize screening of *JAG1*, *NOTCH2*, and of other Notch signaling genes and regulatory regions.

## Supporting information

Supporting information
