# Supplementary material for: Alagille syndrome mutation update: Comprehensive overview of JAG1 and NOTCH2 mutation frequencies and insight into missense variant classification
Source: Hum Mutat. 2019 Aug 26;40(12):2197–220. doi: 10.1002/humu.23879 (PMC6899717; doi:10.1002/humu.23879)
Supplement: Supplementary file 1 — Supporting information [file HUMU-40-2197-s001.pdf]

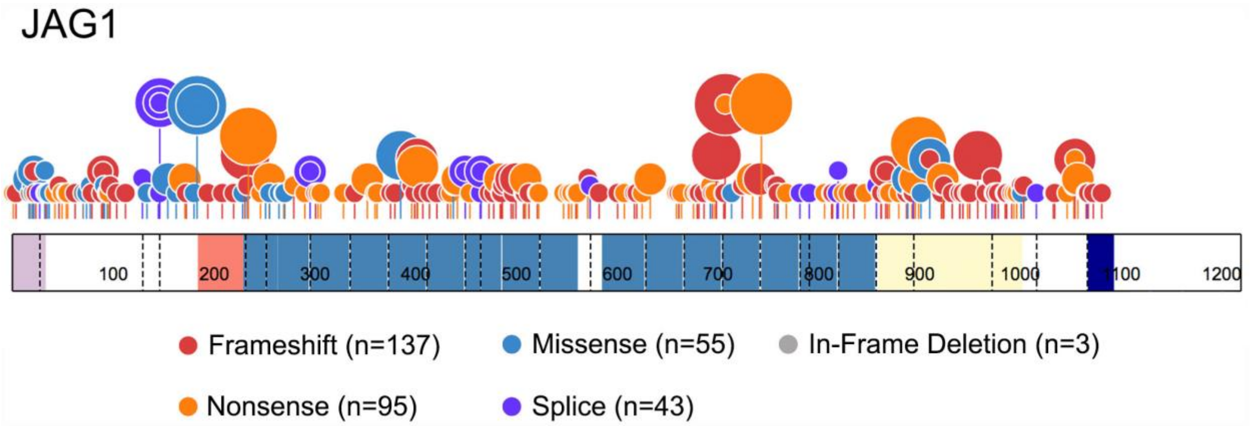

Supporting Information Figure S1. Schematic of all *JAG1* variants found in our single-center study. Variant types are color-coded and the number of probands with each mutation type is indicated. Circle size and height is in proportion to the number of probands with that mutation. Concentric circles indicate different variants that occur at the same locus. Concentric circles of different colors indicate different mutation types that occur at the same locus. Dashed lines within the protein indicate exon boundaries and numbers indicate amino acid coordinates. Protein domains include: signal peptide (lavender), DSL domain (salmon), EGF-like repeats (blue), cysteine-rich domain (yellow), and transmembrane domain (purple). RefSeq NM_000214.2. Images were prepared using ProteinPaint software from Saint Jude Children's Research Hospital–Pediatric Cancer Data Portal (Zhou et. al., 2016).

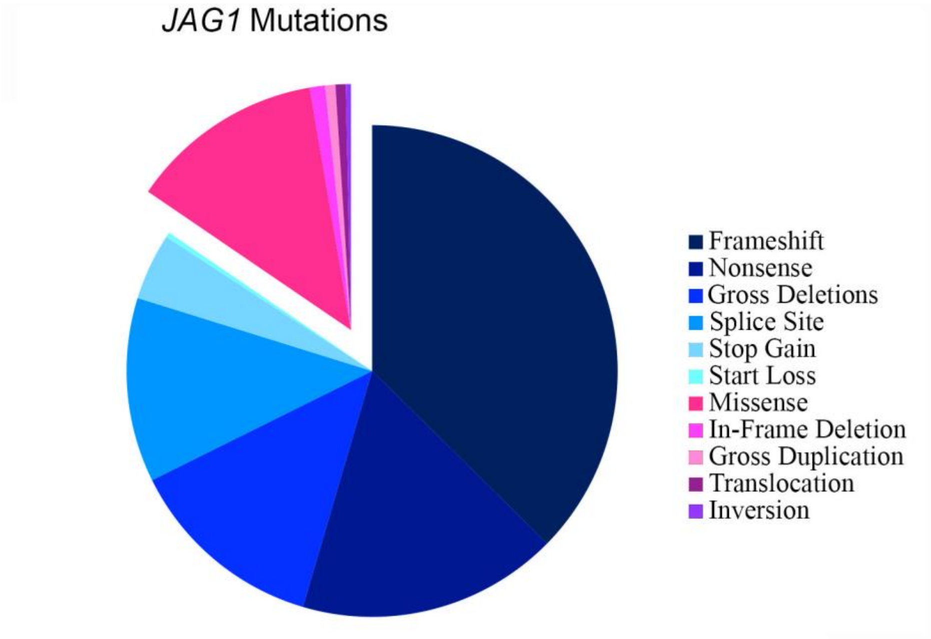

Supporting Information Figure S2. Incidence of *JAG1* mutation types found in our single-center study. Protein-truncating variants are shown in blue color tones and include: frameshift (n=111), nonsense (n=51), splice site (n=36), gross deletion (n=39), stop gain (n=13), and start loss (n=1). Non-protein-truncating variants are shown in pink color tones and include: missense (n=38), in-frame deletion (n=3), gross duplication (n=2), translocation (n=2), and inversion (n=1).

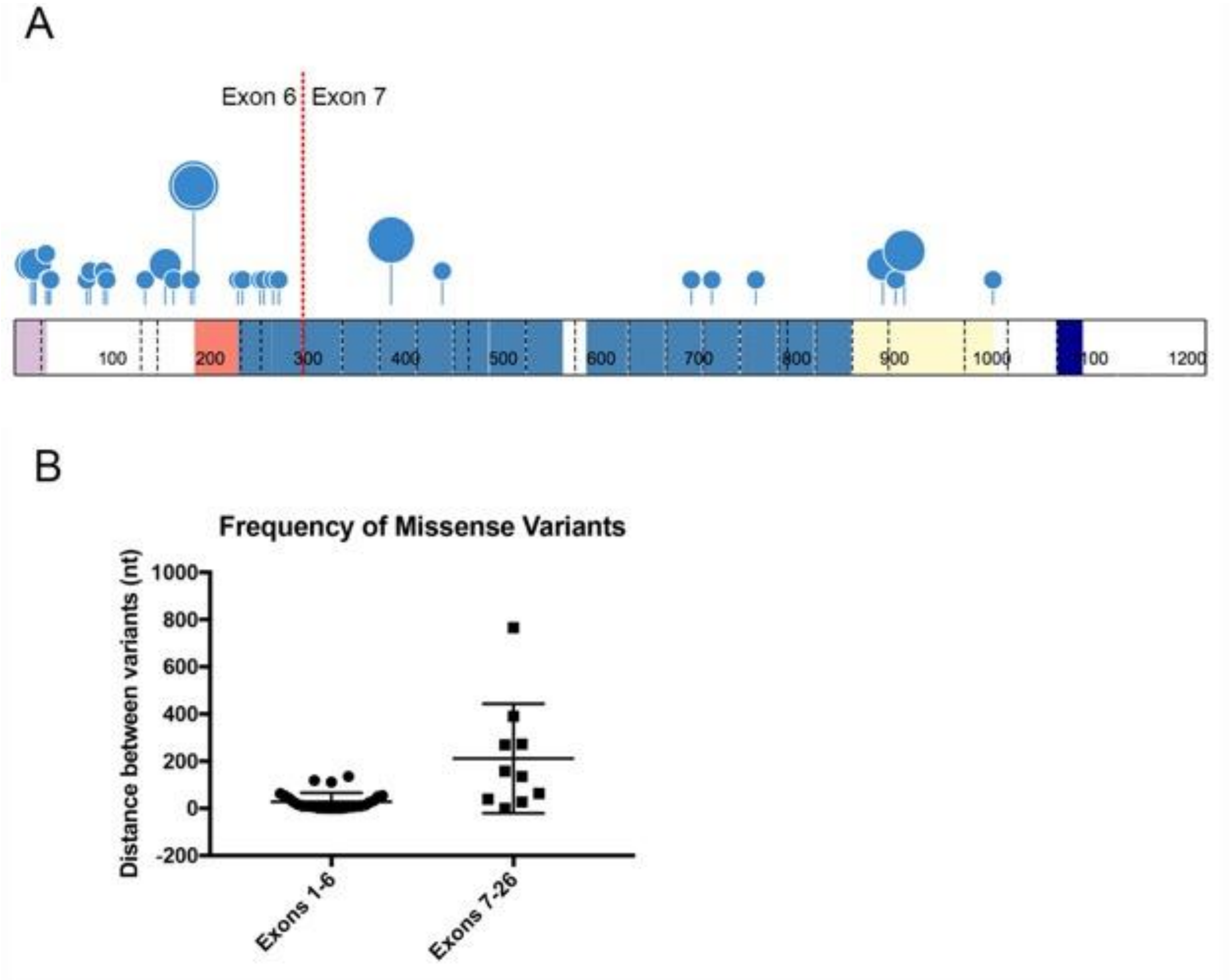

Supporting Information Figure S3. Missense variants are overrepresented in the first 6 exons of *JAG1* in our single-center study. (a) Missense variants from our study are plotted along a protein schematic of *JAG1*. Circle size and height is proportional to the number of probands with each variant. Concentric circles indicate different variants that occur at the same locus. Dashed lines within the protein indicate exon boundaries and numbers indicate amino acid coordinates. (b) The distance in nucleotides was measured between missense variants within exons 1-6 and exons 7-26. Statistical significance (P = 0.0002) was calculated using an unpaired, two-tailed t-test. RefSeq NM_000214.2. Protein schematic was prepared using ProteinPaint software from Saint Jude Children's Research Hospital–Pediatric Cancer Data Portal (Zhou et. al., 2016).

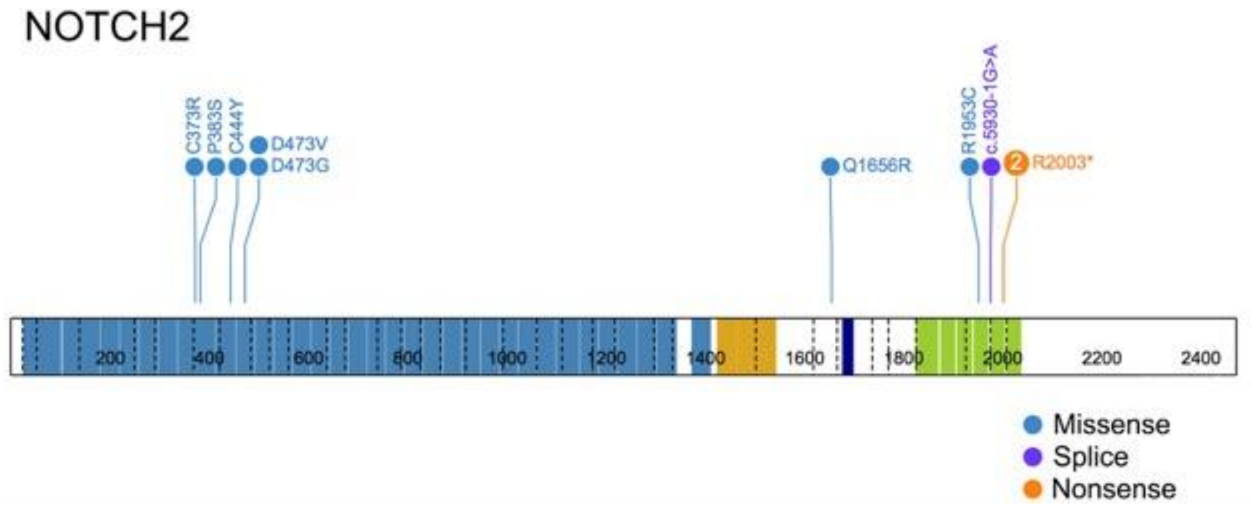

Supporting Information Figure S4. Schematic of all *NOTCH2* variants found in our single-center study. Numbers within each circle indicate the number of probands with that variant. Dashed lines within the protein indicate exon boundaries and numbers indicate amino acid coordinates. Protein domains include: EGF-like repeats (blue), LNR domain (yellow), transmembrane domain (purple), and ANK repeats (green). RefSeq NM_024408.3. Images were prepared using ProteinPaint software from Saint Jude Children's Research Hospital–Pediatric Cancer Data Portal (Zhou et. al., 2016).

Supporting Information Table 1 Clinical features of patients with novel *NOTCH2* pathogenic variants

NOTCH2 Patients	Paucity	Cholestasis	Cardiac	Butterfly	Facies	Eye	Other	Family History
AGS 310-p	No	Yes	Yes<br>PA, VSD	Yes	ND	ND	Duodenal atresia<br>Imperforate anus<br>Renal -single kidney	No
AGS 510-p	Yes	Yes	Yes<br>VSD	ND	Yes	ND	Horseshoe kidney	ND
AGS 510-m	Yes	Yes	No	ND	Yes	ND		ND
AGS 535-p	No	Yes	PFO	No	Maybe	No		Yes - Mat FH of TOF
AGS 535-MA	ND	ND	ND	ND	ND	ND		ND

FH: family history, Mat: maternal, ND: not determined, PA: pulmonary atresia, PFO: patent foramen ovale, TOF: tetralogy of fallot, VSD: ventricular septal defect

Supporting Information Table 2 Clinical features of ALGS patients without *JAG1* or *NOTCH2* pathogenic variants

Mutation Negative Patients	Paucity	Cholestasis	Cardiac	Butterfly	Facies	Eye	Other	Family History
6-p	Yes	Yes	Yes<br>Murmur	No	No	Yes<br>Axenfeld's		
63-p	Yes	Yes	No	Yes	Yes	No	Renal<br>Hydronephrosis	
164-p	Yes	Yes	Yes<br>ASD	Yes	ND	Yes<br>PE		
299-p	Yes	Yes	Yes<br>PS, ASD	No	Atypical-with broad forehead and pointed chin	ND	No renal involvement	Mother with facies
339-p	No	Yes	Yes<br>ASD, AS, PS	No	Yes	Yes<br>PE		
349-p	No	Yes	Yes<br>TOF, PA	Yes	Yes	ND	Renal - VUR surgery<br>Possible cerebral ischemic changes	Mother with facies, PS
398-p	Yes	Yes	Yes<br>PPS, ASD	ND	Yes	ND	Renal - echogenic kidneys	
425-p	No	Yes	Yes<br>PPS	Yes	Yes	Yes<br>PE		
428-p	No	Yes	Yes<br>PPS	Yes	Yes	No	"Bleeding event"<br>Renal - single kidney	
461-p	No	Yes	Yes<br>PPS	No	No (Infant)	Yes<br>PE	Bowel obstruction<br>Vascular event	Mother with acute renal failure in childhood
478-p	Yes	Yes	Yes<br>Mild PA stenosis	No	Atypical-with broad forehead and pointed chin	Yes		
557-p	ND	ND	ND	ND	Yes	ND		ND

621-p	Yes	Yes	No	Yes	Yes	No		

AS: aortic stenosis, ASD: atrial septal defect, ND: not determined, PA: pulmonary atresia, PE: posterior embryotoxon, PPS: peripheral pulmonic stenosis, TOF: tetralogy of fallot, VUR: vesicoureteral reflux

Supporting Information Table 3 Classification of missense variants identified in our study

Exon	DNA Variant	Protein Change	Protein Domain	Probands	Affected Family Members	Novel	Frequency in gnomAD	References	Classification	ACMG Evidence*
1	c.50T>C	p.Leu17Pro	Signal Peptide	2	0	Yes	Not Present		Pathogenic	PS2*, PS4, PM1, PM2, PP3
1	c.59T>C	p.Leu20Pro	Signal Peptide	1	0	No	Not Present	Guegan et. al., 2012	Pathogenic	PS4, PM1, PM2, PM5, PP3
1	c.59T>G	p.Leu20Arg	Signal Peptide	1	2	No	Not Present	Izumi et. al., 2016	Likely Pathogenic	PM1, PM2, PM5, PP1, PP3
1	c.64T>C	p.Cys22Arg	Signal Peptide	2	0	No	Not Present	Lin et. al., 2012	Likely Pathogenic	PS4, PM1, PM2
2	c.97G>A	p.Gly33Ser	Signal Peptide	1	0	No	Not Present	Warthen et. al., 2006	Likely Pathogenic	PM1, PM2, PM5, PM6, PP3
2	c.97G>C	p.Gly33Arg	Signal Peptide	1	0	Yes	Not Present		Pathogenic	PS2, PM1, PM2, PM5, PP3
2	c.98G>A	p.Gly33Asp	Signal Peptide	1	0	No	Not Present	Colliton et. al., 2001	Likely Pathogenic	PM1, PM2, PM5, PP3
2	c.98G>T	p.Gly33Val	Signal Peptide	1	0	No	Not Present	Warthen et. al., 2006	Pathogenic	PS2, PM1, PM2, PM5, PP3
2	c.104T>C	p.Phe35Ser		1	2	Yes	Not Present		Likely Pathogenic	PM1, PM2, PP1, PP3
2	c.110T>C	p.Leu37Ser		1	0	No	Not Present	Colliton et. al., 2001<br>Morrissette et. al., 2001<br>Bauer et. al., 2010	Pathogenic	PS2, PS3, PM1, PM2, PP3
2	c.221A>G	p.Tyr74Cys		1	2	Yes	Not Present		VUS	PM1, PM2, PP1*
2	c.232T>A	p.Cys78Ser		1	0	Yes	Not Present		Pathogenic	PS3, PM1, PM2, PM5<br>• **Functional evidence described in this report (PS3)**
2	c.232T>G	p.Cys78Gly		1	0	No	Not Present	Lin et. al., 2012	Likely Pathogenic	PM1, PM2, PM5
2	c.274T>C	p.Cys92Arg		1	0	No	Not Present	Warthen et. al., 2006	Likely Pathogenic	PM1, PM2, PM5, PP3
2	c.275G>A	p.Cys92Tyr		1	3	No	Not Present	Warthen et. al., 2006	Pathogenic	PS3, PM1, PM2, PM5, PP1, PP3<br>• **Functional evidence described in this report (PS3)**
2	c.283G>C	p.Gly95Arg		1	2	Yes	Not Present		VUS	PM1, PM2, BS4*
3	c.401T>C	p.Leu134Ser		1	0	Yes	Not Present		VUS	PM2, PM5, PP3
4	c.463G>C	p.Ala155Pro		2	0	No	Not Present	Warthen et. al., 2006	Likely Pathogenic	PS4, PM2, PP3
4	c.488C>G	p.Pro163Arg		1	0	No	Not Present	Ropke et. al., 2003	Likely Pathogenic	PS2*, PS4, PM2, PM5
4	c.541T>A	p.Tyr181Asn		1	0	No	Not Present	Colliton et. al., 2001	VUS	PM1, PM2, PP3
4	c.550C>T	p.Arg184Cys		3	0	No	Not Present	Krantz et. al., 1998	Pathogenic	PS2, PS4, PM1, PM2, PM5, PP3
4	c.551G>A	p.Arg184His		7	1	No	Not Present	Krantz et. al., 1998<br>Morrissette et. al., 2001<br>Tada et. al., 2012	Pathogenic	PS2, PS3, PS4, PM1, PM2, PM5, PP1
4	c.686G>A	p.Cys229Tyr	DSL Domain	1	0	No	Not Present	Heritage et. al., 2000	Pathogenic	PS2*, PS3, PM1, PM2, PM5, PP3<br>• **Functional evidence described in this report (PS3)**
5	c.700T>G	p.Cys234Gly	EGF1	1	0	Yes	Not Present		Likely Pathogenic	PM1, PM2, PM5, PP3
5	c.754A>G	p.Arg252Gly	EGF1	1	2	No	Not Present	Warthen et. al., 2006	VUS	PM1, PM2, PP1<br>• ***Predicted change at splice donor site 2 bp downstream****
6	c.766G>A	p.Gly256Ser	EGF1	1	1	No	Not Present	Warthen et. al., 2006	Likely Pathogenic	PM1, PM2, PM5, PP3
6	c.794G>T	p.Cys265Phe	EGF2	1	2	No	Not Present	Guegan et. al., 2012	Pathogenic	PS4, PM1, PM2, PP1*, PP3
6	c.811T>C	p.Cys271Arg	EGF2	1	1	No	Not Present	Warthen et. al., 2006	Pathogenic	PS2*, PS3, PM1, PM2, PP3<br>• **Functional evidence described in this report (PS3)**
9	c.1156G>A	p.Gly386Arg	EGF5	4	7	No	Not Present	Heritage et. al., 2000<br>Tada et. al., 2012	Pathogenic	PVS1, PS4, PM1, PM2, PP1, PP3<br>• Makes a cryptic splice site leading to 37 bp deletion in exon 9*

10	c.1313G>T	p.Cys438Phe	EGF6	1	1	No	Not Present	Crosnier et. al., 1999	Pathogenic	PS3, PS4, PM1, PM2, PM5, PP3<br>• **Functional evidence described in this report (PS3)**
10	c.1313G>C	p.Cys438Ser	EGF6	1	1	Yes	Not Present		Likely Pathogenic	PM1, PM2, PM5, PP3
16	c.2078G>A	p.Cys693Tyr	EGF12	1	1	No	Not Present	Warthen et. al., 2006	Pathogenic	PS3, PM1, PM2, PM6, PP3<br>• **Functional evidence described in this report (PS3)**
17	c.2141G>A	p.Cys714Tyr	EGF13	1	3	No	Not Present	Colliton et. al., 2001 Tada et. al., 2012	Pathogenic	PS3, PM1, PM2, PP1, PP3<br>• **Functional evidence described in this report (PS3)**
18	c.2276G>T	p.Cys759Phe	EGF14	1	1	Yes	Not Present		Likely Pathogenic	PS2*, PM1, PM2, PP3
22	c.2666G>A	p.Arg889Gln	CRD	2	3	No	17/282830 alleles	Warthen et. al., 2006	VUS	PS4, PM1, PP1*
23	c.2705G>C	p.Cys902Ser	CRD	1	3	No	Not Present	Colliton et. al., 2001	Likely Pathogenic	PS3, PM1, PM2, PP1<br>• **Functional evidence described in this report (PS3)**
23	c.2732G>A	p.Cys911Tyr	CRD	3	3	No	Not Present	Warthen et. al., 2006	Pathogenic	PS2*, PS3, PS4, PM1, PM2, PM6, PP3<br>• **Functional evidence described in this report (PS3)**
24	c.3004T>C	p.Cys1002Arg	CRD	1	1	Yes	Not Present		Likely Pathogenic	PS2, PM1, PM2, PP3

RefSeq NM_000214.2

Note: **ACMG Evidence* (Richards et. al., 2015)

Evidence for Pathogenic Impact

Very Strong	PVS1	Null allele in a gene where LOF is a known disease mechanism
Strong	PS1	Same amino acid change as a previously established pathogenic variant regardless of nucleotide change
	PS2	*de novo*
	PS3	Well-established *in vitro* or *in vivo* functional study is supportive of a damaging effect on the gene/gene product
	PS4	The prevalence of the variant in affected individuals is significantly increased compared with the prevalence in controls (seen in multiple probands)
Moderate	PM1	Located in a mutational hot spot and/or critical or well-established functional domain
	PM2	Absent from controls (gnomAD)
	PM5	Novel missense change at an amino acid residue where a different missense change determined to be pathogenic has previously been seen
	PM6	Assumed *de novo* without confirmation of paternity or maternity
Supporting	PP1	Co-segregation with disease in multiple affected family members in a gene definitively known to cause the disease
	PP3	Multiple lines of computational evidence support a deleterious effect on the gene or gene product

Evidence for Benign Impact

Strong	BS1	Allele frequency is greater than expected for disorder
	BS4	Lack of segregation in affected family members
